# Characterization of functional methylomes by next-generation capture sequencing identifies novel disease-associated variants

**DOI:** 10.1038/ncomms8211

**Published:** 2015-05-29

**Authors:** Fiona Allum, Xiaojian Shao, Frédéric Guénard, Marie-Michelle Simon, Stephan Busche, Maxime Caron, John Lambourne, Julie Lessard, Karolina Tandre, Åsa K. Hedman, Tony Kwan, Bing Ge, Lars Rönnblom, Mark I. McCarthy, Panos Deloukas, Todd Richmond, Daniel Burgess, Timothy D. Spector, André Tchernof, Simon Marceau, Mark Lathrop, Marie-Claude Vohl, Tomi Pastinen, Elin Grundberg, The Multiple Tissue Human Expression Resource Consortium, Kourosh R. Ahmadi, Chrysanthi Ainali, Amy Barrett, Veronique Bataille, Jordana T. Bell, Alfonso Buil, Emmanouil T. Dermitzakis, Antigone S. Dimas, Richard Durbin, Daniel Glass, Neelam Hassanali, Catherine Ingle, David Knowles, Maria Krestyaninova, Cecilia M. Lindgren, Christopher E. Lowe, Eshwar Meduri, Paola di Meglio, Josine L. Min, Stephen B. Montgomery, Frank O. Nestle, Alexandra C. Nica, James Nisbet, Stephen O'Rahilly, Leopold Parts, Simon Potter, Johanna Sandling, Magdalena Sekowska, So-Youn Shin, Kerrin S. Small, Nicole Soranzo, Gabriela Surdulescu, Mary E. Travers, Loukia Tsaprouni, Sophia Tsoka, Alicja Wilk, Tsun-Po Yang, Krina T. Zondervan

**Affiliations:** 1 Department of Human Genetics, McGill University, 740 Docteur-Penfield Avenue, Montreal, Québec , Canada H3A 0G1; 2 McGill University and Genome Quebec Innovation Centre, 740 Docteur-Penfield Avenue, Montreal, Québec, Canada H3A 0G1; 3 Institute of Nutrition and Functional Foods (INAF), Université Laval, 2440 Hochelaga Boulevard, Québec, Québec, Canada G1V 0A6; 4 Québec Heart and Lung Institute, Université Laval, 2725 Sainte-Foy Road, Québec, Québec, Canada G1V 4G5; 5 Department of Medical Sciences, Uppsala University, Akademiska sjukhuset Ingång 40, Uppsala 75185, Sweden; 6 Department of Medical Sciences, Molecular Epidemiology, Uppsala University, Dag Hammarskjölds väg 14B, Uppsala 75185, Sweden; 7 Science for Life Laboratory, Uppsala University, Dag Hammarskjölds väg 14B, Uppsala 75185, Sweden; 8 Wellcome Trust Centre for Human Genetics, University of Oxford, Roosevelt Drive, Oxford OX3 7BN, UK; 9 Oxford Centre for Diabetes, Endocrinology and Metabolism, University of Oxford, Churchill Hospital, Headington, Oxford OX3 7JU, UK; 10 Oxford National Institute for Health Research Biomedical Research Centre, Churchill Hospital, Headington, Oxford OX3 7JU, UK; 11 Wellcome Trust Sanger Institute, Wellcome Trust Genome Campus, Hinxton, Cambridge CB10 1SA, UK; 12 William Harvey Research Institute, Barts and The London School of Medicine and Dentistry, Queen Mary University of London, Charterhouse Square, London EC1M 6BQ, UK; 13 Roche NimbleGen, 500 South Rosa Road, Madison, Wisconsin 53719, USA; 14 Department of Twin Research and Genetic Epidemiology, King's College London, St Thomas' Campus, Lambeth Palace Road, London SE17EH, UK; 15 Department of Informatics, School of Natural and Mathematical Sciences, King's College London, Strand, London WC2R 2LS, UK; 16 Department of Genetic Medicine and Development, University of Geneva Medical School, Geneva 1211, Switzerland; 17 University of Cambridge, Cambridge CB3 0WA, UK; 18 European Bioinformatics Institute, Hinxton CB10 1SD, UK; 19 University of Cambridge Metabolic Research Labs, Institute of Metabolic Science Addenbrooke's Hospital Cambridge CB2 0QQ, UK; 20 Cambridge National Institute for Health Research Biomedical Research Centre, Addenbrooke's Hospital, Cambridge CB2 0QQ, UK; 21 St. John's Institute of Dermatology, King's College London, London SE1 9RT, UK.

## Abstract

Most genome-wide methylation studies (EWAS) of multifactorial disease traits use targeted arrays or enrichment methodologies preferentially covering CpG-dense regions, to characterize sufficiently large samples. To overcome this limitation, we present here a new customizable, cost-effective approach, methylC-capture sequencing (MCC-Seq), for sequencing functional methylomes, while simultaneously providing genetic variation information. To illustrate MCC-Seq, we use whole-genome bisulfite sequencing on adipose tissue (AT) samples and public databases to design AT-specific panels. We establish its efficiency for high-density interrogation of methylome variability by systematic comparisons with other approaches and demonstrate its applicability by identifying novel methylation variation within enhancers strongly correlated to plasma triglyceride and HDL-cholesterol, including at *CD36*. Our more comprehensive AT panel assesses tissue methylation and genotypes in parallel at ∼4 and ∼3 M sites, respectively. Our study demonstrates that MCC-Seq provides comparable accuracy to alternative approaches but enables more efficient cataloguing of functional and disease-relevant epigenetic and genetic variants for large-scale EWAS.

## Abstract (web summary)

Currently, genome-wide methylation studies are limited to using targeted arrays or enrichment to assess large sample sizes. Here, Allum *et al*. demonstrate MethylC-Capture Sequencing, a cost-effective method for investigating genetic and epigenetic variation.

## Graphical abstract

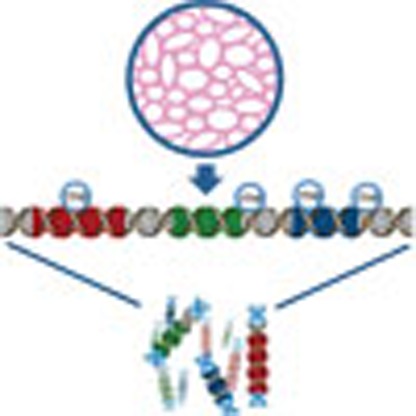

DNA methylation is an epigenetic modification that was previously thought to be important only for gene silencing through hypermethylation of CpG islands in promoter regions; however, recent studies have revealed more diverse functions dependent on genomic location^1^. For instance, hypermethylation within the gene bodies is likely to be indicative of primed expression and is associated with increased gene expression^2^,^3^. Profiling of histone modifications by chromatin immunoprecipitation and high-throughput sequencing (ChIP-Seq) has uncovered strong correlations between chromatin structure and DNA methylation, with hypomethylated regions associated to active marks or open chromatin and hypermethylated regions suggestive of repressed regulatory regions and heterochromatin^2^. H3K4me3, known to mark nucleosomes flanking transcription start sites and CpG-rich promoters, is negatively associated with DNA methylation, whereas distal regulatory elements (that is, enhancers) marked by H3K4me1 are relatively CpG poor with a more variable hypo- to hemimethylated profile^3^. As the majority of approaches assessing the human methylation landscape have been biased to CpG-rich regions^3^, the methylation pattern of enhancers remains to be described in more detail.

Other investigated features of DNA methylation variation in human populations include the effects of environmental^4^ and genetic factors^3^,^5^, and the role of methylation in complex disease susceptibility^6^,^7^,^8^. We recently estimated methylation levels of ∼450,000 CpGs in subcutaneous adipose tissue (AT) across 648 female twins and identified common sequence variants that contribute significantly to methylation variability, with indications that some of these variants may also mediate genetic risk for metabolic diseases^3^ possibly through changes in methylation levels. We further noted that in these variables, disease-linked methylation sites are enriched in distal regulatory elements, paralleling earlier findings of common sequence variants identified in genome-wide association studies (GWAS) being enriched in active chromatin measured by DNaseI hypersensitivity^9^ or within tissue-specific enhancer marks^10^. In addition, the most comprehensive study to date of methylation profiles across multiple tissues also highlighted that enhancers contain tissue-specific variable CpGs that co-localize with tissue-specific transcription factors^11^.

However, the majority of methylation quantitative trait loci (QTL) and epigenome-wide association studies (EWAS) presented to date have used the Illumina Human Methylation 450 BeadChip array (Illumina 450K array). Although covering ∼480,000 CpGs in the human genome, the Illumina 450K array is biased towards regions with high CpG content such as promoters, which we and others have demonstrated to have limited inter-individual and inter-tissue variation^3^,^11^. Largely due to the invariable state of promoter-located CpGs, these regions are also known to be depleted among significant disease-associated sites^3^. Importantly, tissue-specific and disease-relevant regions such as enhancers are greatly underrepresented on the Illumina 450K array.

In contrast to available targeted methodologies^12^ or alternative sequencing methods biased towards CpG-dense regions such as reduced representation bisulfite sequencing (RRBS) or methylated DNA immunoprecipitation, whole-genome bisulfite sequencing (WGBS) allows complete characterization of the methylation landscape. However, with only ∼20% or less of CpGs being variable across individuals or tissues^11^, WGBS is inefficient for large-scale population studies, as it has a high cost and requires in-depth sequencing capacity to achieve sufficient coverage. Thus, none of the above methods are optimal for comprehensive studies of methylation variation and their impact on complex diseases. Alternative approaches to interrogate functional (that is, regulatory active) methylomes are needed for more comprehensive yet cost-effective identification of biologically relevant CpGs associated to complex diseases.

Here, we present methylC-capture sequencing (MCC-Seq), a next-generation sequencing capture approach interrogating functional methylomes in disease-targeted tissues or cells. We design AT-specific panels to probe up to ∼4.5 × 10^6^ putative functional DNA methylation sites as defined by their localization to hypomethylated footprints and regulatory elements, as well as ∼2.8 × 10^6^ single-nucleotide polymorphisms (SNPs) for simultaneous genotyping profiling. We validate the method through comparisons with WGBS, Illumina 450K array and Agilent SureSelect Human Methyl-Seq (Agilent SureSelect) data, and show that MCC-Seq yields comparable resolution over targeted intervals. We demonstrate the ability of MCC-Seq to identify novel biologically relevant epigenetic variants associated to disease by applying the panel in a disease–trait association study of 72 individuals. Our initial results illustrate the advantages of this new approach over currently used methods for methylome analysis, providing a viable alternative for powerful and cost-effective large-scale interrogation of functional methylomes.

## Results

### First-generation capture panel design for MCC-Seq

Using human AT as a model, we designed a first-generation sequence panel to capture the putative functional and disease-linked methylome in AT (Met V1) (Table 1 and Methods). We targeted 87 Mb of sequence comprising (1) hypomethylated footprints, generated from WGBS data of 30 AT samples, (2) regulatory elements (identified by H3K4me1 and H3K4me3 ChIP-Seq) in human adipocytes characterized by the NIH Roadmap Epigenomics Mapping Consortium and (3) ∼50 K CpGs with known association to metabolic phenotypes^3^ (Supplementary Data 1). All together, the panel targets 2,496,975 unique CpGs with 210,883 directly overlapping Illumina 450K array-targeted CpGs. By including both putative enhancer and promoter regions, we aimed to obtain a more complete profile of AT-specific regulatory regions and to investigate the variability status of these CpGs at increased depth over previous studies^3^.

In MCC-Seq, a whole-genome sequencing library is prepared, bisulfite converted and amplified, followed by a capture enriched for targeted bisulfite-converted DNA fragments (Methods). This is achieved through the novel SeqCap Epi probe design platform by Roche NimbleGen, which enables capture of double-stranded targets regardless of their methylated state via high tiling density of probes. To test the efficiency and performance of Met V1, we performed targeted enrichment of both uniplex (1-plex) and multiplexed library samples (2-plex, 4-plex, 6-plex and 10-plex). Each capture was sequenced on a single lane of the 100 bp paired-end Illumina HiSeq2000/2500 System. Generated reads were aligned to the converted reference genome using BWA v.0.6.1 (ref. 13) and filtered according to our benchmark bioinformatics workflow (Methods) using a read depth cutoff per CpG of ≥5X.

The sequence statistics obtained for the different captured pooled samples are summarized in Supplementary Table 1. The average on-target CpG read depth ranged from 13X (10-plex) to 82X (1-plex) and the percentage of total reads that mapped within the target CpGs averaged 62% (ranging from 51% to 80%), and was independent of the degree of multiplexing. The average number of targeted CpGs with ≥5X depth of sequence coverage decreased with increasing multiplexing from 94% (1-plex) to 63% (10-plex) of targeted CpGs (Supplementary Table 1).

### Second-generation panel design for comprehensive profiling

Based on the performance of the first AT-specific panel, we developed and assessed a comprehensive second-generation AT MCC-Seq panel (Met V2) that encompasses additional AT-specific regulatory regions and variants, and additional SNPs throughout the genome for simultaneous methylation and genetic association studies (Table 1 and Methods). The Met V2 panel targets 156 Mb of sequence spanning 4,442,383 unique CpGs and 2,840,815 autosomal biallelic SNPs from dbSNP 137. The regions covered by the Met V2 panel include the following: (1) CpGs contained within low (LMRs) and unmethylated regions (UMRs) identified from merged data sets of 30 WGBS AT samples (Supplementary Data 2); (2) CpGs located within human adipocyte regulatory elements (H3K4me1 and H3K4me3) from the NIH Roadmap Epigenomics Mapping Consortium; (3) all 482,421 CpGs from the Illumina 450K array; (4) 28,947 regions covering metabolic disease-associated GWAS loci from the National Human Genome Research Institute GWAS catalogue (9 January 2014); and (5) the 256,327 SNPs from the Illumina HumanCore BeadChip.

To assess the performance of the larger Met V2 panel, we applied the MCC-Seq protocol and performed targeted enrichment on a 6-plex capture. Sequencing was conducted on one lane of the 100-bp paired-end Illumina HiSeq2000/2500 System. On average, 62% of the reads mapped to target regions with 15X mean coverage and 65% of the target regions were covered at a sequence depth of ≥5X (Supplementary Table 2).

### Sample-based validation of MCC-Seq

**Figure 1:**
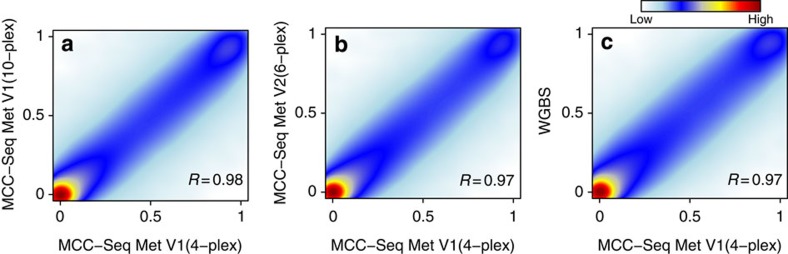
Technical replication of MCC-Seq methylation calls and comparison with WGBS. Correlation between technical replicates from a DNA sample derived from VAT sequenced from independent captures (**a**) of the same MCC-Seq sequence panel (Met V1) (4-plex and 10-plex; *N*=1,587,026 CpGs; *R*=0.98) and (**b**) of two different MCC-Seq sequence panels (Met V1_4-plex_ and Met V2_6-plex_; *N*=1,569,170 CpGs; *R*=0.97). (**c**) Comparison between MCC-Seq_4-plex_ and WGBS (*N*=1,620,874 CpGs; *R*=0.97) methylation calls for the same VAT DNA sample. Only CpGs with sequence coverage ≥5X in MCC-Seq and WGBS experiments were included in the analysis; *R* is the Pearson's correlation coefficient.

As a first validation step, we assessed the effects of technical variability on methylation profiles by comparing the results obtained from a single DNA sample derived from visceral AT (VAT) prepared in replicate experiments with independent captures on the same panel (Met V1) and different degrees of multiplexing (4-plex versus 10-plex). We found a high concordance of the methylation calls for overlapping CpGs (*N*=1,587,026; average coverage_4-plex_=36X; average coverage_10-plex_=30X; *R*=0.98; Pearson's correlation is used throughout; Fig. 1a). We also assessed technical variability of the methylation calls by comparing the results from a different DNA sample prepared in replicate experiments with independent captures using the two different panels and different degrees of multiplexing (Met V1_4-plex_ versus Met V2_6-plex_), and confirmed the high concordance in methylation calls for CpGs (*N*=1,569,170; average coverage_MetV1(4-plex)_=16X; average coverage_MetV2(6-plex)_=30X; *R*=0.97; Fig. 1b).

**Figure 2:**
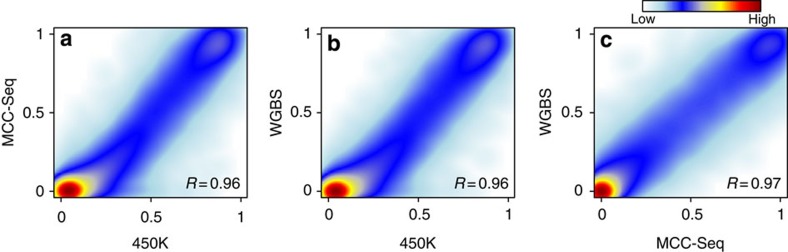
Comparison of methylation calls obtained with different methods. (**a**) Correlation between MCC-Seq_4-plex_ and Illumina 450K array methylation calls for the same VAT DNA sample (*R*=0.96), (**b**) comparison between WGBS and Illumina 450K array results (*R*=0.96) and (**c**) comparison between WGBS and MCC-Seq_4-plex_ results (*R*=0.97). Only CpGs with data available from all three techniques were included (*N*=150,898 CpGs); we required sequence coverage ≥5X for MCC-Seq and WGBS; *R* is the Pearson's correlation coefficient.

Next, we compared MCC-Seq methylation with WGBS data from the same sample. Here we also found a high correlation (MCC-Seq versus WGBS) for overlapping CpGs (*N*=1,620,874; average coverage_MCC-Seq_=31X; average coverage_WGBS_=23X; *R*=0.97; Fig. 1c). In addition, we evaluated the sequence results against the Illumina 450K array data on the subset of these CpGs that are included in the array by comparing this method against both MCC-Seq and WGBS. For both comparisons, we obtained correlations of *R*=0.96 (*N*=150,898; average coverage_MCC-Seq_=32X; average coverage_WGBS_=23X; Fig. 2 and Supplementary Table 3). To rule out any biases in the comparisons, we also restricted the correlations to CpGs with intermediate methylation levels by excluding completely hypo- (0%) and hypermethylated (100%) CpGs based on the WGBS and MCC-Seq data. Encouragingly, we found the high correlation being maintained with *R*=0.95 (*N*=45,097; average coverage_MCC-Seq_=33X) and *R*=0.94 (*N*=45,097; average coverage_WGBS_=25X) for MCC-Seq versus Illumina 450K and WGBS versus Illumina 450K, respectively (Supplementary Fig. 1). Using this limited set of CpGs profiles across multiple approaches we were also able to confirm the importance of generating sufficient sequence depth for accurate methylation calls, as correlation was shown to improve with increased read-depth cutoffs (Supplementary Table 4). Similar improvement in correlations of methylation calls by MCC-Seq and Illumina 450K was seen with increasing read depth (Supplementary Fig. 2).

Finally, we contrasted methylation calls from MCC-Seq against Agilent SureSelect—another targeted-sequencing approach based on a different methylation capture strategy than described here, allowing only single-strand capture of smaller target regions, and thus not suitable for comprehensive genotype profiling. More specifically, MCC-Seq relies on the efficient capture of targeted methylated and unmethylated CpGs (up to 160 Mb or 4.4 M CpGs) in bisulfite-converted libraries, whereas Agilent SureSelect captures target regions before bisulfite conversion and requires larger amounts of input DNA. By juxtaposing both capture approaches using the same sample sequenced at extreme depth, we obtained correlations that mimic those of our technical replicates shown above (*N*=2,551,186; average coverage_SureSelect_=137X; average coverage_MCC-Seq_=216X; *R*=0.99; Supplementary Fig. 3A). This high correlation (*N*=1,734,371; average coverage_SureSelect_=156X; average coverage_SureSelect_=230X; *R*=0.99; Supplementary Fig. 3B) was also seen when excluding completely hypo- (0%) and hypermethylated (100%) CpGs in both approaches, indicating accuracy in measurement for CpGs with intermediate methylation levels as shown above.

### Population-based validation of MCC-Seq

We then applied MCC-Seq Met V1 to a set of 72 VAT samples derived from obese individuals (body mass index (BMI) >40 kg m^−2^) aged 19–67 years, undergoing bariatric surgery and diagnosed with or without metabolic syndrome^14^ (Methods). Metabolic syndrome was diagnosed when individuals had abdominal obesity and at least two of the following four criteria set by the National Cholesterol Education Program Adult Treatment panel III^15^: elevated plasma fasting glucose, high triglyceride (TG) levels, high blood pressure or lower high-density lipoprotein cholesterol (HDL-C) levels. Using the 4-plex pooling approach, we sequenced the samples to an average read depth of 25X for on-target CpGs. At a sequence depth of ≥5X, a total of 2,147,576 CpGs were detected in at least one individual, with 1,882,222 (88%) CpGs detected in at least 50% of the samples. In all subsequent population-based analyses, we required ≥5X coverage based on our comparisons described above (Supplementary Fig. 2). In addition to requiring ≥5X coverage, we eliminated CpGs that had low coverage, by removing those that were below the 20th percentile for averaged coverage over the 72 samples for the distribution across all CpGs. This yielded 1,710,209 CpGs for further consideration (Supplementary Fig. 4 and Methods) with an average sequence depth of 30X and a minimum of 18X. An outline of all population-based analyses is shown in Supplementary Fig. 5.

First, we characterized the methylation pattern of these 1.7 M CpGs assessed within 72 AT samples and noted that, as expected, the majority (69%) of the captured CpGs exhibited a hypomethylated pattern (defined as <20% methylation) with only 17% being hemi- to hypermethylated (defined as >50% methylation; Supplementary Fig. 6). We also characterized these CpGs by assessing their genomic localization within putative regulatory regions through their overlap with histone marks (H3K4me1 and H3K4me3) in human adipocytes and hypomethylated footprints from our WGBS on 30 AT samples (Methods). To do this, we first characterized hypomethylated footprints by distinguishing between LMRs and UMRs in the WGBS data as previously described^16^ (Methods and Supplementary Data 2). We noted that LMRs were associated with CpG-poor distal regulatory regions (average methylation level of 24%), whereas UMRs are CpG-dense and mapped principally to promoter regions (average methylation level of 9%; Supplementary Fig. 7). For the regulatory elements overlapping H3K4me3 marks (active promoters), we restricted our analysis to locations within 1 kb of transcription start sites of known RefSeq transcripts and not overlapping H3K4me1 marks as previously described^3^. We then assessed the population variability of methylation levels for CpGs mapping to H3K4me1 marks or LMRs (putative enhancers) and compared this with similar estimates of methylation variation for CpGs mapping to H3K4me3 marks or UMRs (putative promoter regions). As previously reported^3^, methylation of CpGs that map to enhancer elements are more variable across individuals (median s.d.=9.4), whereas promoter regions display a more invariable pattern (median s.d.=1.5; Supplementary Fig. 8).

We then profiled a subset (*N*=24) of the 72 VAT samples (Supplementary Fig. 5) with the Illumina 450K array, for direct comparisons of methylation scores estimated by the two methods when considering multiple samples. We applied a normalization method on the Illumina 450K array data to reduce technical biases that have been shown to have an impact on the *β*-values^17^ (Methods). The average correlation of methylation levels estimated by the two methods was *R*=0.50 and *R*=0.58, respectively, for the top 25% (*N*=34,517, median s.d.=11.0) and top 10% (*N*=13,807; median s.d.=13.6) most variable CpGs in the MCC-Seq data based on s.d. estimates of each CpGs (Supplementary Fig. 9). These population-based correlations of MCC-Seq versus the Illumina 450K array are noticeably lower than the sample-based correlations described above; however, given the different nature of the comparisons, that is, correlation of the methylation measurements at each CpG in multiple individuals here versus the overall correlation across all CpGs within a single sample, they cannot be directly compared. As such, we find that the sample-based correlations across the 24 samples are similar to that described above for a single sample, ranging from *R*=0.93 to 0.96.

### Population-based genotype profiling by MCC-Seq

The same 24 AT samples described above were also genotyped with the Illumina HumanOmni2.5S-8 BeadChip array for validation of MCC-Seq's ability to simultaneously call genotypes. After stringent quality control, we obtained SNP genotypes at 94,600 overlapping loci using MCC-Seq (Met V1) (Methods). We observed 99% genotype concordance between the two methods at sites on the SNP array, indicating that MCC-Seq has the potential to allow for simultaneous and accurate genotyping calling over regions of interest. Similarly, comparing the observed heterozygosity from the two measurements yielded high correlation (Supplementary Fig. 10).

In total and based on dbSNP 137, we determined that the Met V1 panel has the potential to detect 1,343,928 autosomal biallelic SNPs within its target regions, of which an average of 1,300,369 (97%) per sample were covered at a read depth of ≥5X. In the broader Met V2 panel, there is a heightened potential for autosomal biallelic SNP detection (2,840,815) with an average of 2,666,458 (94%) SNPs detected per sample at 5X read coverage. Thus, the performance of the Met V2 panel is similar to that of the V1 panel, despite its more extensive coverage (forexample, 156 versus 87 Mb).

### EWAS of TG levels using MCC-Seq

**Figure 3:**
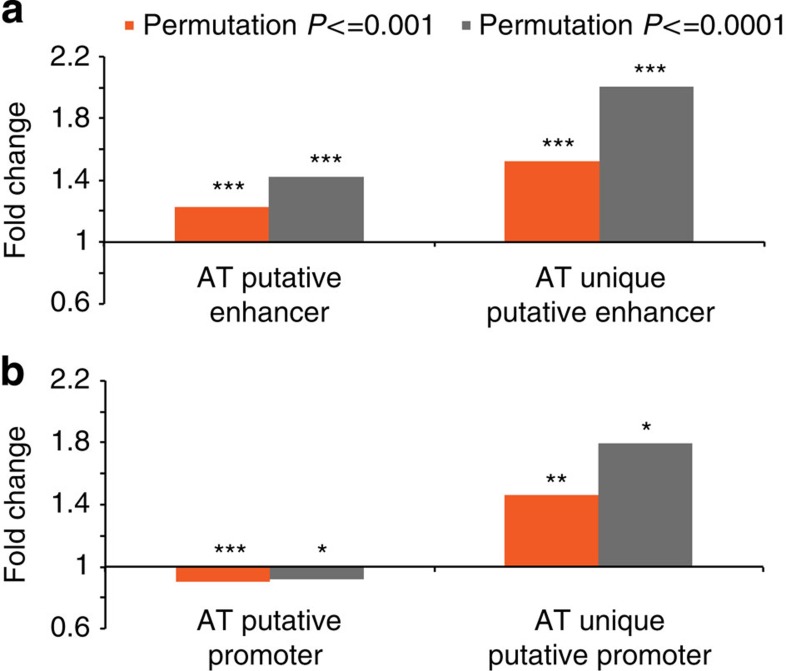
Annotation of TG-associated CpGs in putative regulatory regions. CpGs with average reads coverage above the 20th percentile that showed evidence of association with TG (*P*≤0.001 or *P*≤0.0001) were annotated with additional data. (**a**) This panel shows significant enrichment (*y* axis, fold-change) of TG-associated CpGs for *P*≤0.001 (orange bars) and *P*≤0.0001 (grey bars) within putative enhancer regions as defined by H3K4me1 marks and/or LMRs (^***^P=5.3 × 10^−7^ for *P*≤0.001 and *P*=4.9 × 10^−5^ for *P*≤0.0001 CpGs, respectively), and for H3K4me1 marks and/or LMRs unique to AT (^***^P=6.0 × 10^−10^ for *P*≤0.001 and *P*=4.1 × 10^−7^ for *P*≤0.0001 CpGs, respectively). (**b**) This panel shows significant depletion (*y* axis, fold-change) of the same TG-associated CpGs significance (*P*≤0.001 shown as orange bars and *P*≤0.0001 shown as grey) within putative promoter regions as demarcated by H3K4me3 marks and/or UMRs (^***^P=7.1 × 10^−10^ for CpGs with *P*≤0.001 and *P=0.023 for CpGs with *P*≤0.0001), but enrichment when restricting to H3K4me3 marks and/or UMRs unique to AT (^**^P=2.4 × 10^−3^ for CpGs with *P*≤0.001 and *P=0.020 for CpGs with *P*≤0.0001). Enrichment was established using Fisher's exact test.

To illustrate the application of MCC-Seq for epigenome mapping of a quantitative trait, we examined plasma TG levels measured on the 72 individuals for which the MCC-Seq Met V1 data were available. We note that TG exhibits substantial individual variability in the study cohort (Supplementary Fig. 11). To assess associations, we applied a generalized linear model (GLM) assuming a binomial distribution of methylation levels and adjusting for BMI, age and biological sex along with the sequence depth at each CpG. We assigned a nominal significance for the trait association using a permutation test (Methods). We identified 2,580 CpGs with *P*-value ≤0.001 (Supplementary Data 3) and 518 CpGs with *P*-value ≤0.0001. The locations of these potential TG-associated CpGs were evaluated with respect to putative regulatory regions through their overlap with histone marks (H3K4me1 and H3K4me3) in human adipocytes, and LMRs and UMRs identified as described above (Methods). As shown in Fig. 3a, TG-associated CpGs (*P*≤0.001) were found to map preferentially to H3K4me1 (enhancer) histone marks and/or LMRs (Fisher's exact test *P*=5.3 × 10^−7^). This pattern was even more pronounced when information on LMRs unique to AT and H3K4me1 peaks was combined (Methods) to demarcate putative enhancers (Fisher's exact test *P*=6.0 × 10^−10^). This supports the mounting evidence that disease–trait-associated epigenetic variants localize, to a large extent, to distal regulatory regions. Similar results were also observed when restricting the analysis to CpGs that met the more stringent criterion of *P*≤0.0001 in the permutation test (Fig. 3a). Furthermore, at both *P*-value cutoffs, we observed depletion of TG-associated CpGs within putative promoter regions that are shared across tissues as detected by either H3K4me3 histone marks or UMRs (Fisher's exact test *P*=7.1 × 10^−10^) versus enrichment when restricting to promoter marks unique to AT (Fisher's exact test *P*=2.4 × 10^−3^; Fig. 3b).

We further examined the subset of MCC-Seq TG-associated CpGs that overlapped nearby (250 bp flanking the CpG) CpGs from the Illumina 450K array used in an independent cohort of ∼650 female individuals from the MuTHER study^3^ with TG measurements and AT samples available. MuTHER is a population-based cohort study that includes female twins (1/3 dizygotic and 2/3 monozygotic) aged 38.7–84.6 years recruited from the TwinsUK resource^18^, which has previously been shown to be comparable to population singletons in terms of disease-related and lifestyle characteristics^19^. Methylation data on AT samples from the study members that were previously profiled on the lllumina 450K array were tested for association with TG levels using a linear mixed model similar to the above but also incorporating familial relationship, twin zygosity and other cofactors (Methods). We selected the top TG-associated MuTHER CpGs within the 250 bp of the 2,580 tested CpGs identified at *P*≤0.001 (Supplementary Data 3), revealing 1,582 Illumina 450K array CpGs. Of these, 124 (8%) were found to be significantly associated with TG (Bonferroni *P*-value threshold of *P*=0.05; nominal *P*-value: 0.05/1,582=3.2 × 10^−5^) in the MuTHER data with the same direction of effect as observed in our study (8.9-fold enrichment; binomial test, *P*<2.2 × 10^−16^). Encouragingly, the replication was strengthened when limiting to Illumina 450K array CpGs directly overlapping the 2,580 TG-associated MCC-Seq CpGs with 18 out of 171 sites (11%) significantly associated in MuTHER with the same direction of effect as in MCC-Seq results (18.0-fold enrichment; binomial test, *P*<2.2 × 10^−16^).

### Assessment of loci harbouring TG-associated CpGs

We further annotated the most significant TG-associated CpGs (*P*≤0.0001) mapping to an AT-specific regulatory element (*N*=89; Fig. 3) in further detail (Supplementary Data 4). First, we assessed their association to TG in the larger MuTHER cohort as described above. In total, 33/89 TG-associated CpGs overlapped a nearby Illumina 450K-measured CpG (250 bp flanking the CpG) and were included in the analysis. Here, 21% were found to also be significantly associated with TG in the MuTHER cohort, with the same direction of effect using the stringent Bonferroni *P*-value threshold of *P*=0.05 as estimated above; nominal *P*-value=3.2 × 10^−5^. Furthermore, using the nominal *P*-value of 0.05 and the same direction of effect, as many as 16 CpGs (48%) showed evidence of association to TG in the independent cohort (Supplementary Table 5).

We recently showed high degree of sequence dependency of AT DNA methylation and thus also examined the potential existence of genetic regulation among the TG-associated CpGs using our publicly available *cis*-mQTL data from AT profiled on the Illumina 450K array^3^. Again, we used the 33 CpGs that overlapped a nearby Illumina 450K as described above. We found that methylation levels of 55% of these TG-associated CpGs are regulated by a nearby SNP at 1% false-discovery rate^3^ representing twofold enrichment (Fisher's exact test *P*=0.0017), indicating that a large proportion of trait-associated epigenetic variants identified here are under genetic control (Supplementary Table 5).

Next, we used transposase-accessible chromatin sequencing (ATAC-Seq), as detailed in the Methods, on adipocyte nuclei isolated from AT of an obese individual undergoing bariatric surgery, to further pinpoint and fine-map the effects of the TG-associated CpGs linked to hypomethylated footprints. ATAC-Seq^20^ is an antibody-independent method for profiling active regulatory regions by mapping open chromatin with sensitivity that is comparable to DNaseI-Seq but with the advantage of requiring only ∼100,000 input cells. Here we found that 65/89 CpGs (73%) were nearby (within 250 bp) or directly overlapping an ATAC-Seq peak, indicating that these CpGs could be mapped with high confidence to active regulatory regions in pure adipocytes.

We further examined the expression pattern of genes in the vicinity of our top CpGs in human adipocytes compared with various blood cell types (Methods). Candidate genes were identified as overlapping or within 100 kb of the TG-associated CpGs. We performed RNA sequencing (RNA-Seq) of adipocyte nuclei isolated from visceral and subcutaneous AT of four obese individuals undergoing bariatric surgery matching our discovery cohort, as well as from B cells, T cells and monocytes of four healthy donors (Methods). Differential expression analysis (Methods and Supplementary Data 5) revealed 38/76 CpGs (50%) being associated with genes significantly more expressed in adipocytes (when comparing both visceral- and subcutaneous-derived expression, log2 fold change>2, *P*<0.05) which is a significant enrichment of adipocyte-specific expression (1.6-fold, Fisher's exact test, *P*=6.6 × 10^−4^).

We also examined the overlap of our potential TG-associated loci with the National Human Genome Research Institute catalogue of results from GWAS (accessed January 2014) and found that genes linked to 19 (23%) of our CpGs were previously cited for a metabolic disease trait based on GWAS (1.5-fold enrichment; Fisher's exact test, *P*=0.06; Supplementary Data 5). These genes include *CD36* (HDL-C), *RPTOR* (obesity) and *ABCG5/ABCG8* (low-density lipoprotein cholesterol (LDL-C) and total cholesterol). Additional follow-up data on *CD36* is provided below.

### Follow-up of the TG-associated loci mapping to *CD36*

**Figure 4:**
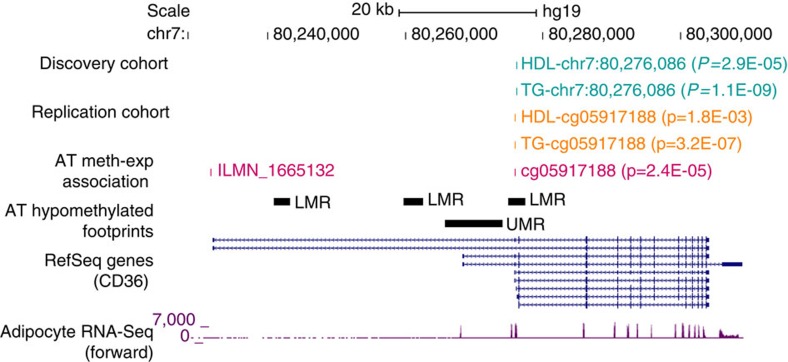
Top TG-associated CpG mapping to an AT-specific regulatory region—*CD36*. The top TG-associated CpG (chr7:80,276,086-80,276,087; *P*=1.1 × 10^−9^; GLM assuming a binomial distribution; turquoise track) identified in the discovery cohort maps within an intragenic region of *CD36*, which overlaps an AT-unique LMR (black track). Investigation within a population-based cohort (*N*∼650) replicated the epigenetic effect in a nearby 450K array probes (orange track); mapping to the same regulatory region (cg05917188; *P*=3.2 × 10^−7^; linear mixed model). The methylation status of the latter probe was also found to be negatively associated to *CD36* expression in AT (ILMN_1665132; *P*=6.7 × 10^−5^; linear mixed model, pink track). AT-specific expression of the gene was also noted through AT RNA-Seq data (purple track).

To illustrate these results, we selected the most significant CpG of the TG-associated loci for additional follow-up studies (chr7:80,276,086-80,276,087; GLM *P*=1.1 × 10^−9^; Fig. 4 and Supplementary Data 4). This CpG is located within an intragenic region of *CD36*, a gene encoding a glycoprotein with an important role in lipid metabolism^21^,^22^ that has been linked to metabolic disease susceptibility^23^. Levels of circulating CD36 protein were recently reported to be positively correlated to plasma TG levels in obese individuals^24^ and SNPs near the gene were associated to HDL-C levels in a large GWAS^25^. The TG-associated CpG maps to an LMR unique to AT. Using RNA-Seq data generated from both human adipocytes derived from obese individuals and blood cells from healthy controls as described above (Supplementary Data 5), we found significantly higher *CD36* expression in adipocytes than in blood cells (GLM, log2 fold change=2.4–11.0, *P*=6.3 × 10^−22^–3.3 × 10^−161^). In an attempt to study whether the potential enhancer region where the TG-associated CpG maps controls expression of *CD36*, we used our publicly available array-based expression (IlluminaHT12) and methylation (Illumina 450K array) data from the MuTHER cohort (*N*∼650)^3^. We found that methylation of the closest Illumina 450K array CpG (cg05917188; Fig. 4) was negatively associated with expression of the main *CD36* transcript in AT (linear mixed model, *P*=2.4 × 10^−5^), highlighting a gene regulatory effect of our TG-associated hypomethylated region. Finally, we also used the MuTHER cohort (*N*∼650) and cg05917188 as described above, for validation of the TG association where we were able to verify the pronounced effect of methylation at the regulatory region on TG levels (linear mixed model, *P*=3.2 × 10^−7^; Fig. 4 and Supplementary Table 5). As recent GWAS efforts show links to HDL-C, we also tested for this association to CpG methylation within our discovery cohort and found a similar pattern (GLM, *P*=2.93 × 10^−5^) with concordant results from the MuTHER cohort (linear mixed model, *P*=1.8 × 10^−3^; Fig. 4).

Taken together with the other results described above, our data provide strong evidence in favour of an epigenetic effect of the AT-specific regulatory region of *CD36* on multiple metabolic disease-related traits.

## Discussion

The assessment of DNA methylation has emerged as an essential tool for understanding the aetiology of human disease^26^. Recent reports show that variable and functional epigenetic variants are enriched in enhancers, rather than in promoter and CpG island regions^3^, which are the principal regions assayed by commonly used targeted approaches (for example, Illumina 450K array and RRBS). Although WGBS is comprehensive, it is inefficient for the large-scale investigations that are required for methylation QTL studies and EWAS of common multifactorial diseases. This motivated us to look for an improved method for high-resolution interrogation of the variable functional component of the methylome.

We established MCC-Seq to assess target regions of the genome in a cost-effective and accurate manner. With MCC-Seq, we can examine active regulatory regions in disease-appropriate tissues, specifically permitting us to identify disease-linked DNA methylation variants that are not identifiable with previous targeting approaches. MCC-Seq can include up to 200 Mb in custom, user-defined interrogation panels, which is an advantage over other available capture approaches. Samples can be multiplexed to obtain lower sequencing costs for large-scale EWAS. Although upfront analysis time is needed for proper selection of CpGs, the customizable and flexible design allows easy elimination of CpGs that are invariable across individuals^11^, providing further savings at the sequencing and computational levels. As an example, our Met V2 adipose-specific panel covers ∼4.5 × 10^6^ CpGs in regulatory regions and also includes the complete Illumina 450K panel of ∼480,000 CpGs, allowing comparisons or replication with studies that use the latter. We also demonstrate the capacity for multifunctional assays providing both comprehensive methylome and SNP genotype data, thereby permitting additional data integration than in other techniques such as Agilent SureSelect where single-strand capture bias inhibits complete genotype profiling. As such, the Met V2 panel includes the complete set of SNPs from the Illumina HumanCore BeadChip, which covers highly informative genome-wide tag SNPs found across globally diverse populations, allowing for further high-density genotype imputation.

Comparisons of MCC-Seq to three alternative approaches—WGBS, Illumina 450K array and Agilent SureSelect—indicated that methylation calls derived from MCC-Seq correlated highly with all three methods (for example, *R*>0.96) with Illumina 450K array showing slightly lower correlation. The lower correlation of MCC-Seq and WGBS with the Illumina 450K array data may be attributable to technical differences in DNA methylation assessment (that is, microarray versus next-generation sequencing) and proabably higher specificity of methylation profiles called from sequencing methods at sufficient depth. Overall, we believe that MCC-Seq with its larger flexible platform, genotyping ability and low DNA input requirements, is more adapted to large-scale EWAS studies than other studied approaches.

Based on our results, we predict that MCC-Seq will be particularly valuable for the identification of functional, disease-linked DNA methylation variants. In fact, we demonstrated the potential of such an approach by applying an AT-specific panel to a cohort of 72 individuals in which we had measured metabolic-related traits, including TG. In agreement with the current literature, the analysis of the TG-associated CpGs revealed a clear significant enrichment within putative enhancer regions as defined by hypomethylated regions and adipocyte-specific ChIP-Seq data (NIH Roadmap Epigenomics Mapping Consortium) and a clear underrepresentation within putative tissue-independent promoter regions. This demonstrates the importance of investigating putative enhancer regions for functional epigenetic variant identification—regions currently underrepresented in the commonly used Illumina 450K array and RRBS approaches. When comparing the subset of results from MCC-Seq that were available from the Illumina 450K array data on the large MuTHER/TwinsUK cohort of 650 female twins^3^, we found a significant overlap of CpGs exhibiting significant TG association in the two data sets, further validating MCC-Seq as a tool for powerful discovery of trait-associated methylation variation.

Additional investigations were performed for the most significant TG-associated CpG mapping to a regulatory region within *CD36*, which is known to function in fatty acid and glucose metabolism^21^,^22^. Here we were not only able to validate the results in the MuTHER resource showing consistent direction of effect of the association of methylation variation in the regulatory region with TG but also show evidence of regulation of *CD36* expression by our identified AT-specific regulatory region at the population level. These results of a potential AT regulation of *CD36* expression were further strengthened by RNA-Seq data of purified adipocytes and multiple blood cell types showing pronounced difference in the expression pattern with adipocytes expressing *CD36* at considerably higher level. As our discovery cohort included obese individuals diagnosed with or without metabolic syndrome, we also tested another trait used for the diagnosis, HDL-C, in association with DNA methylation at our CpG of interest. Interestingly, we noted a similar association to HDL-C, which was further validated in the MuTHER cohort, indicating that epigenetic variants of *CD36* may be able to serve as a biomarker for cardiovascular disease prediction in obese individuals similar to what has been suggested for circulating plasma *CD36* for type 2 diabetes prediction^27^.

In conclusion, MCC-Seq provides high-resolution and cost-effective interrogation of functional methylomes in disease-relevant tissues with concurrent genotyping of potentially millions of SNPs. With its customizable panel design, our approach permits flexibility in both size and regions, to be interrogated for disease-associated epigenetic variant discovery. Our results demonstrate the significant utility of the approach over WGBS, Illumina 450K array and Agilent SureSelect methods. We demonstrate that targeting active regulatory regions for disease-associated DNA methylation CpG investigation is a valid strategy over whole-genome investigation. Our data suggest that applying MCC-Seq in large cohorts will be a powerful approach to identify trait-associated methylation in studies of human disease.

## Methods

### First-generation panel design

We designed a first-generation capture panel (Met V1) targeting the functional methylome in human AT. Regions incorporated in the panel design included hypomethylated windows generated from merged WGBS data from 30 subcutaneous AT samples derived from the MuTHER/TwinsUK cohort^3^. Briefly, the mean methylation levels per CpG were kept for those detected in at least 3 individuals resulting in 15,462,376 CpGs. We then calculated the probability of obtaining the specific methylation level (excluding complete hypomethylation corresponding to 0%) per CpG in our merged data set. The probabilities were then merged for three, four, five and ten consecutive CpGs within a window of 1 kb. As the majority of CpGs are hypermethylated with a mean methylation of ∼80%, hypomethylated windows corresponded to small probability estimates. Based on these probabilities, we then selected the bottom 2% of the different windows generated for inclusion in the Met V1 panel design.

Next, AT-specific regulatory elements were incorporated into the panel design. Regulatory elements (H3K4me1 and H3K4me3) from AT nuclei derived from five independent donors were downloaded from the NIH Roadmap Epigenomics Project as follows^3^,^28^. Aligned ChIP-Seq reads (BAM files) of the H3K4me1 and H3K4me3 marks, as well as the ChIP-Seq input, were downloaded from the NIH Roadmap Epigenomics Project (GEO repository accessions GSM621425, GSM669908, GSM669975, GSM670045, GSM772757, GSM621435, GSM669925, GSM669988, GSM669998, GSM670041, GSM621401, GSM669934, GSM669940, GSM669984 and GSM670043). Each file of the H3K4me1 and H3K4me3 marks was segmented into 100 bp bins. Within each bin, the sequence reads were counted. The bin counts were divided by the total number of sequence reads to obtain normalized intensity signals. ChIP-Seq input reads were processed in the same way and their normalized signal intensity values were subsequently subtracted from the normalized bin intensity signals. The H3K4me1 and H3K4me3 bins were then ranked according to these values. Based on the mean ranking across the five individuals, the top 1% bins per histone mark were then included in the panel design.

Finally, 53,638 Illumina 450K array probes with CpGs showing association (per-trait Bonferroni *P*<0.05; nominal *P*<1.4.0 × 10^−7^) to metabolic phenotypes (for example, BMI, total cholesterol, HDL-C, LDL-C and total TGs) were selected for inclusion in the Met V1 panel design (Supplementary Data 1). These associations were identified through an analysis of Ilumina 450K array AT methylation data collected from 648 female twins from the MuTHER/TwinsUK resource^3^.

In total, 79.6 Mb of sequence was targeted. Roche NimbleGen R&D was responsible for probe design. Each targeted region was extended to a minimum size of 100 bp and the capture probes were extended beyond the edge of each target to assure coverage yielding a total of 87.3 Mb of sequence in the final panel, which covered 99.2% of our input sequence (Supplementary Data 6). Only 978 of our selected targets failed in the custom probe design. In total, the Met V1 panel targeted 2,496,975 CpGs of which 210,883 overlapped with Illumina 450K array sites.

### Generation of second-generation panel

The second-generation panel for adipose methylome capture (Met V2) was designed to cover 131 Mb including extension to 100 bp and additional flanking regions. We identified and incorporated into the panel design AT hypomethylated regions as described under ‘Identification of hypomethylated regions'. Inclusion was limited to UMRs below a size of 7,000 bp and LMRs above 100 bp (excluding two large outliers; Supplementary Data 2). Selected hypomethylated regions covered 2,213,942 and 469,962 CpGs for UMRs and LMRs, respectively. Similar as in Met V1, AT regulatory regions were also incorporated into the panel design, selecting the 677,809 and 1,327,121 CpGs from the top 1% bins of regulatory elements (H3K4me1 and H3K4me3) characterized in human adipocytes by the NIH Epigenome Roadmap consortium as described above. Furthermore, we included all 482,421 CpGs on the Illumina 450K array and all 256,327 SNPs from the Illumina HumanCore SNPs. Finally, we selected 28,947 metabolic disease GWAS SNPs from the GWAS catalogue for inclusion into the panel design.

We merged all selected regions using the R/Bioconductor package GenomicRanges. Roche NimbleGen generated a 156.2-Mb panel based on our regions, covering 97.9% of our original targeted sequences in 629,845 regions (Supplementary Data 7). Summary of the generated panel indicated that that 16,759 of our original targets were unsuccessfully covered by the custom probes. We determined that the maximum CpG coverage capacity of the Met V2 panel is 4,442,383 CpGs.

### MCC-Seq protocol

The MCC-Seq protocol was developed and optimized in collaboration with Roche NimbleGen R&D. Briefly, in MCC-Seq a whole-genome sequencing library is prepared and bisulfite converted, amplified and a capture enriching for targeted bisulfite-converted DNA fragments is carried out. The captured DNA is further amplified and sequenced. More specifically, whole-genome sequencing libraries were generated from 700 to 1,000 ng of genomic DNA spiked with 0.1% (w/w) unmethylated *λ* DNA (Promega) previously fragmented to 300–400 bp peak sizes using the Covaris focused-ultrasonicator E210. Fragment size was controlled on a Bioanalyzer DNA 1000 Chip (Agilent) and the KAPA High Throughput Library Preparation Kit (KAPA Biosystems) was applied. End repair of the generated dsDNA with 3′- or 5′-overhangs, adenylation of 3′-ends, adaptor ligation and clean-up steps were carried out as per KAPA Biosystems' recommendations. The cleaned-up ligation product was then analysed on a Bioanalyzer High Sensitivity DNA Chip (Agilent) and quantified by PicoGreen (Life Technologies). Samples were then bisulfite converted using the Epitect Fast DNA Bisulfite Kit (Qiagen), according to the manufacturer's protocol. Bisulfite-converted DNA was quantified using OliGreen (Life Technologies) and, based on quantity, amplified by 9–12 cycles of PCR using the Kapa Hifi Uracil+DNA polymerase (KAPA Biosystems), according to the manufacturer's protocol. The amplified libraries were purified using Ampure Beads and validated on Bioanalyzer High Sensitivity DNA Chips, and quantified by PicoGreen.

Next, SeqCap Epi Enrichment System protocol (Roche NimbleGen) was carried out for the capture. The hybridization procedure of the amplified bisulfite-converted library was performed as described by the manufacturer, using 1 μg of total input of library, which was evenly divided by the libraries to be multiplexed, and incubated at 47 °C for 72 h. Washing and recovering of the captured library, as well as PCR amplification and final purification, were carried out as recommended by the manufacturer. Quality, concentration and size distribution of the captured library was determined by Bioanalyzer High Sensitivity DNA Chips. Each capture was sequenced on the Illumina HiSeq2000/2500 system using 100 bp paired-end sequencing.

### MCC-Seq methylation profiling

Reads were aligned to the bisulfite-converted reference genome using BWA v.0.6.1 (ref. 13). We removed the following: (i) clonal reads, (ii) reads with low mapping quality score (<20), (iii) reads with >2% mismatch to converted reference over the alignment length, (iv) reads mapping on the forward and reverse strand of the bisulfite-converted genome, (v) read pairs not mapped at the expected distance based on library insert size and (vi) read pairs that mapped in the wrong direction as described by Johnson *et al*.^29^. To avoid potential biases in downstream analyses, CpGs were further filtered as follows: CpGs not covered by at least five reads, CpGs not covered by at least two reads per strand, CpGs overlapping an SNP (dbSNP 137) and sites overlapping DAC Blacklisted Regions or Duke Excluded Regions generated by the ENCODE project:

(http://hgwdev.cse.ucsc.edu/cgi-bin/hgFileUi?db=hg19&g=wgEncodeMapability).

We further selected CpGs sites that exhibited ≤20% methylation difference between strands. Finally, all off-target reads were removed. Methylation values at each site were calculated as total (forward and reverse) non-converted C-reads over total (forward and reverse) reads. CpGs were included in subsequent analysis if the number of sequence reads was five or greater. In some analyses, we also excluded sites at which the average sequence depth over all study individuals was below the 20th percentile in the complete data set. CpGs were counted once per location combining both strands together.

### Illumina 450K array methylation profiling

Bisulfite conversion was conducted on 1 μg of a subset of 24 VAT DNA samples and quantitative DNA methylation analysis was carried out at the McGill University and Génome Québec Innovation Centre (Montreal, Canada). Infinium HumanMethylation450 BeadChip (Illumina) was processed according to the manufacturer's instructions. Methylation data were visualized and analysed using the GenomeStudio software version 2011.1 (Illumina) and the Methylation Module. None of the samples were excluded following quality control steps assessed by bisulfite conversion, extension, staining, hybridization, target removal, negative and nonpolymorphic control probes. Methylation levels (*β*-values) were estimated as the ratio of signal intensity of the methylated alleles to the sum of methylated and unmethylated intensity signals of the alleles (*β*-value=C/(T+C)). The *β*-values vary from 0 (no methylation) to 1 (100% methylation). Methylation *β*-values were further quantile normalized to remove unwanted technical variation, using control probes as recently presented^17^.

### Agilent SureSelect CpG profiling and MCC-Seq comparisons

An MCC-Seq panel (Roche NimbleGen) was designed to mimic the SureSelect Human Methyl-Seq panel (Agilent) by designing probes against the same genomic coordinates, but targeting both DNA strands. As the MCC-Seq protocol hybridizes probes to library fragments after bisulfite treatment and PCR amplification, when the sequences of those fragments may be highly variable depending on the CpG density and initial methylation status of each CpG within each original DNA molecule, multiple probes with different sequences were designed to permit effective hybridization capacity over the full range of possible post-bisulfite sequences. The MCC-Seq and SureSelect Methyl-Seq capture experiments were executed at Roche NimbleGen (Madison, WI), while the SureSelect Methyl-Seq captures and sequencing were performed by a third-party service provider, according to manufacturer's instructions, using 1 μg (MCC-Seq) or 3 μg (SureSelect) of DNA extracted from the LCL GM12878 cell line.

MCC-Seq reads were filtered according to our bioinformatics pipeline described above (MCC-Seq methylation profiling). Given the single-strand bias of the Agilent method, no filters were applied on the Agilent SureSelect data. Comparisons of the methylation calls from both methods were made for overlapping sites at ≥5X (*N*=2,551,186 CpGs) and at ≥10X (*N*=2,496,975 CpGs).

### Trait-association discovery cohort

Between June 2000 and July 2012, 1,906 severely obese men (*N*=597) and women (*N*=1,309) undergoing biliopancreatic diversion with duodenal switch^30^ at the Quebec Heart and Lung Institute (Quebec City, Quebec, Canada) were recruited. Subjects had fasted overnight before the surgical procedure. Anaesthesia was induced by a short-acting barbiturate and maintained by fentanyl and a mixture of oxygen and nitrous oxide. VAT samples were obtained within 30 min of the beginning of the surgery from the greater omentum^31^. Here, a subset of the VAT cohort was included corresponding to 72 individuals (BMI >40 kg m^−2^; discovery cohort) free of metabolic diseases such as type 2 diabetes, cardiomyopathy, or endocrine disorders. Thirty-five individuals were deemed to have metabolic syndrome (MetS+ group), while the remaining 37 were not affected (MetS− group). The presence of MetS was determined by the National Cholesterol Education Program Adult Treatment Panel III guidelines when an individual fulfilled three or more criteria^15^. None of the study participations was on medication to treat MetS features. The sample collection of AT was approved by the Université Laval and McGill University (IRB FWA00004545) ethics committee and performed in accordance with the principles of the Declaration of Helsinki. Tissue banking and the severely obese cohort were approved by the research ethics committees of the Quebec Heart and Lung Institute. All participants provided written informed consent before enrolment in the study.

Body weight, height, waist girth and resting systolic and diastolic blood pressure were measured preoperatively by standardized procedures. BMI was calculated as weight in kilograms divided by height in metres squared. Plasma total cholesterol (total-C), TG and HDL-C levels were measured using enzymatic assays. HDL-C was measured in the supernatant following precipitation of very-low-density lipoproteins and low-density lipoproteins with dextran sulphate and magnesium chloride. Plasma LDL-C levels were estimated with the Friedewald formula. Fasting glucose concentrations were enzymatically measured^32^.

### DNA isolation

Genomic DNA was extracted from 200 mg of all 72 VAT samples using the DNeasy Blood & Tissue kit (Qiagen), as recommended by the manufacturer, and quantified using both NanoDrop Spectrophotometer (Thermo Scientific) and PicoGreen DNA methods.

### Identification of hypomethylated regions

We merged WGBS data from 30 healthy individuals, filtered as described under ‘MCC-Seq methylation profiling', to define AT-specific hypomethylated regulatory regions. A minimum of three individuals per CpG was set as a threshold for inclusion into the merged set. We applied the R/Bioconductor package MethylSeekR to the data set, to identify and define regulatory regions as LMRs and UMRs. Briefly, this package uses a cutoff method wherein UMRs and LMRs are predicted at single-base resolution as regions of consecutive CpGs having methylation statuses under a set level with UMRs being differentiated from LMRs based on a minimum content of 30 CpGs^16^. By default, a methylation threshold of 50% and false-discovery rate of 5% was set for the analysis^16^, fixing consecutive CpGs at ≥4. We identified 20,195 UMRs and 45,065 LMRs for AT. The same procedure was carried out in WGBS data collected from whole-blood samples of the same cohort, identifying 19,871 UMRs and 46,159 LMRs. We intersected the AT and whole-blood hypomethylated regions and found 2,342 and 24,687 AT-unique UMRs and LMRs, respectively.

### Genotyping

The same samples (*N*=24) included for Illumina 450K methylation profiling were also selected for high-density genotyping using the Illumina HumanOmni2.5-8 (Omni2.5) BeadChip according to protocols recommended by Illumina. After applying quality control filters, genotypes were retrained for 2,132,665 SNP sites. Simultaneous genotypes calls from MCC-Seq data (Met V1) were inferred using the Bis-SNP^33^ software, a bisulfite-sequencing variant caller, with default parameters: ‘-T BisulfiteGenotyper -stand_call_conf 20 -stand_emit_conf 0 -mmq 30 -mbq 17 -minConv 0' and with dbSNP 137 as prior SNP information. The aligned bam files were used as input file and the hg19 was used as the reference genome. These genotypes were then compared with the genotypes from HumanOmni-2.5 M genotyping data.

### Epigenome-wide association of TG levels

Associations of methylation levels of CpGs detected in VAT (*N*=72) with TG levels were tested using a GLM function implemented in R3.1.1. Two outliers in TG levels were identified by setting a cutoff of mean±3*s.d. and removed from any further analysis. The response variable (methylation levels) was fitted to a binomial distribution weighted for sequence read coverage at each site and adjusted for age, sex and BMI. All CpGs associated with TG at *P*<0.05 were subjected to permutation tests, to establish the significance of phenotype effect as follows: the DNA methylation values for each CpG were permuted 10,000 times and the GLM was fitted at each permutation round. Permutation *P*-values were established by counting how many times the permuted association resulted in significance smaller than the observed GLM *P*-value for each CpG. Replication of the 2,580 MCC-Seq TG-associated CpGs with permutation *P*≤0.001 was conducted in AT methylation data from an independent cohort of 648 female individuals in the MuTHER cohort. Associations between Illumina 450K array methylation data and TG levels were assessed using a linear mixed model taking into account familial relationship, twin zygosity and other cofactors (that is, age, beadchip, bisulfite conversion efficiency and bisulfite-treated DNA input) and summary statistics were obtained from http://www.sanger.ac.uk/resources/software/genevar/. Expanding to 250 bp flanking regions around MCC-Seq TG-associated sites, we were able to assess replication status in 1,582 sites. Expression QTL data available from this same cohort was further used to validate the TG association established within the MCC-Seq data at the *CD36* loci (http://www.sanger.ac.uk/resources/software/genevar/).

### Adipocyte nuclei isolation

Subcutaneous and visceral adipose tissues were collected from obese individuals undergoing biliopancreatic diversion with duodenal switch. Mature adipocytes were isolated as follows^34^: freshly sampled adipose tissues were minced and digested in Krebs Ringer Henseleit Buffer (1 M HEPES, 2 M NaCl, 1 M KCl, 1 M CaCl_2_, 1 M MgCl_2_, 1 M K_2_HPO_4_, pH 7.4) supplemented with 5 mM glucose, 0.1 μM adenosine, 0.1 mg ml^−1^ ascorbic acid, 4% electrophoresis grade, delipidated BSA and 350 U ml^−1^ collagenase (Worthington Biochemical Corp., Lakewood, NJ) for 45 min with agitation (37 °C). Adipocyte suspensions were filtered through nylon mesh and washed with the buffer. Isolated adipocytes were homogenized in two volumes of lysis buffer (25 mM Tris pH 7.5, 5 mM MgCl_2_, 0.5% Triton X-100, 0.3 M sucrose and protease inhibitors) for 2 min on ice, then centrifuged at 3,220*g* for 25 min (4 °C). The pellets were washed twice with lysis buffer and resuspended in nuclei storage buffer (50 mM Tris pH 7.8, 5 mM MgCl_2_, 0.1 mM EDTA, 0.1 mM dithiothreitol, 40%v/v glycerol) for freezing.

### Transposase-accessible chromatin sequencing

ATAC-Seq libraries were generated on 100,000 mature adipocyte nuclei using a modified protocol to that published recently^20^. More precisely, transposase reaction was carried out for 30 min at 37 °C in a 25-μl reaction volume using 10X transposase concentration (Illumina Nextera Kit). EDTA (25 mM) was added to the reaction mix and transferred to ice before recovering DNA using MinElute PCR Purification columns (Qiagen). Next, samples were PCR enriched (ten cycles; Supplementary Table 6) and DNA was isolated using GeneRead Purification columns (Qiagen). Libraries were quantified by quantitative PCR (Supplementary Table 7), Picogreen and LabChip, then were sequenced on the Illumina HiSeq2500 pair-ended 100 bp, using the Nextera sequencing primers.

Raw reads were trimmed for quality (phred33 ≥30) and length (*n≥*32), and Illumina adapters were clipped off using Trimmomatic v. 0.22 (ref. 35). Filtered reads were aligned to the hg19 human reference using BWA v.0.6.1 (ref. 13). Peaks were called without a control using MACS v. 2.0.10.07132012 (ref. 36) at a *q*-value cutoff of 0.05.

### Blood cell isolation

Peripheral blood mononuclear cells were purified from buffy coats originating from 450 ml blood of healthy blood donors (Uppsala Blood Transfusion Center, Uppsala University Hospital, Sweden), using Ficoll-Paque (GE Healthcare) density-gradient centrifugation. B cells, T cells and monocytes were isolated from dedicated batches of peripheral blood mononuclear cells, using positive selection with CD19+, CD3+ and CD14+ beads (Miltenyi Biotec), respectively, according to the manufacturer's instructions.

### RNA sequencing

RNA isolations was performed using miRNeasy Mini Kit (Qiagen). RNA library preparations were carried out on 500 ng of RNA with RNA integrity number (RIN)>7 isolated from adipocyte cells extracted from AT^37^,^38^ and blood cells (CD19+, CD3+ and CD14+) using the Illumina TruSeq Stranded Total RNA Sample preparation kit, according to manufacturer's protocol. Final libraries were analysed on a Bioanalyzer and sequenced on the Illumina HiSeq2000 (pair-ended 100 bp sequences). Raw reads were trimmed for quality (phred33≥30) and length (*n*≥32), and Illumina adapters were clipped off using Trimmomatic v. 0.32 (ref. 35). Filtered reads were aligned to the hg19 human reference using Tophat v.2.0.10 (ref. 39) and bowtie v.2.1.0 (ref. 40). Raw read counts of UCSC genes were obtained using htseq-count v.0.6.1 (http://www-huber.embl.de/users/anders/HTSeq). Differential expression analysis was done using DESeq^41^ including adipocytes isolated from AT (subcutaneous and visceral) of four obese individuals undergoing bariatric surgery and different blood cell types (B cells, T cells and monocytes) of four healthy individuals (Uppsala Blood Transfusion Center, Uppsala University Hospital, Sweden).

## Additional information

**Accession codes:** The methylation 450K data has been deposited in the Gene Expression Omnibus (GEO), http://www.ncbi.nlm.nih.gov/geo (accession no. GSE59524). All MCC-Seq data from the discovery cohort as well as adipocyte ATAC-Seq and RNA-Seq data can be visualized in the UCSC Genome Browser, http://genome.ucsc.edu, using the Track Hub Data feature (‘McGill Adipose Tissue Epigenome') by adding the following URL to ‘My Hubs': http://hubs.hpc.mcgill.ca/~elin/Adipose_MCCSeq_Hub.txt. All processed MCC-Seq data from the discovery cohort and from the adipocyte RNA-Seq analyses are available in the ArrayExpress database (www.ebi.ac.uk/arrayexpress) accession no. E-MTAB-3181 and E-MTAB-3182). Raw reads from RNA-Seq, ATAC-Seq and MCC-Seq are deposited to the European Genome-phenome Archive (EGA) and available after approval by the Data Access Committee (DAC) designated to the study (https://www.ebi.ac.uk/ega/home).

**How to cite this article:** Allum, F. *et al*. Characterization of functional methylomes by next-generation capture sequencing identifies novel disease-associated variants. *Nat. Commun*. 6:7211 doi: 10.1038/ncomms8211 (2015).

## Supplementary Material

Supplementary Information Supplementary Figures 1-11 and Supplementary Tables 1-7

Supplementary Data 1 Metabolic trait-associated Illumina 450K array CpGs included in the Met V1 design

Supplementary Data 2 AT-specific hypomethylated footprints

Supplementary Data 3 TG-associated CpGs with permutation p-value=0.001

Supplementary Data 4 Annotation of the top TG-associated CpGs

Supplementary Data 5 Differential expression analysis and GWAS association of candidate genes

Supplementary Data 6 Met V1 sequence targets

Supplementary Data 7 Met V2 sequence targets

## Figures and Tables

**Table 1:** Composition of Met V1 and Met V2 panels.

Panel components	Met V1	Met V2
AT-hypomethylated footprints CpGs (*N*)	1,089,355	2,683,904
AT-regulatory elements (H3K4me1 and me3) CpGs (*N*)	1,625,328	1,625,328
Illumina 450K CpGs (*N*)	210,883	482,421
Metabolic trait-associated SNPs (*N*)	—	28,947
Core SNPs (*N*)	—	256,327
Total covered regions (Mb)	87.0	156.2
Total covered CpGs (*N*)	2,496,975	4,442,383
Total covered SNPs (*N*)	1,343,928	2,840,815

SNP, single-nucleotide polymorphism.

## References

[b1] Jones P. A. Functions of DNA methylation: islands, start sites, gene bodies and beyond. Nat. Rev. Genet. 13, 484–492 (2012). 22641018 10.1038/nrg3230

[b2] Consortium, E. P.. . An integrated encyclopedia of DNA elements in the human genome. Nature 489, 57–74 (2012). 22955616 10.1038/nature11247 PMC3439153

[b3] Grundberg E. . Global analysis of DNA methylation variation in adipose tissue from twins reveals links to disease-associated variants in distal regulatory elements. Am. J. Hum. Genet. 93, 876–890 (2013). 24183450 10.1016/j.ajhg.2013.10.004 PMC3824131

[b4] Breitling L. P., Yang R., Korn B., Burwinkel B. & Brenner H. Tobacco-smoking-related differential DNA methylation: 27K discovery and replication. Am. J. Hum. Genet. 88, 450–457 (2011). 21457905 10.1016/j.ajhg.2011.03.003 PMC3071918

[b5] Wagner J. R. . The relationship between DNA methylation, genetic and expression inter-individual variation in untransformed human fibroblasts. Genome Biol. 15, R37 (2014). 24555846 10.1186/gb-2014-15-2-r37 PMC4053980

[b6] Dayeh T. . Genome-wide DNA methylation analysis of human pancreatic islets from type 2 diabetic and non-diabetic donors identifies candidate genes that influence insulin secretion. PLoS Genet. 10, e1004160 (2014). 24603685 10.1371/journal.pgen.1004160 PMC3945174

[b7] Liu Y. . Epigenome-wide association data implicate DNA methylation as an intermediary of genetic risk in rheumatoid arthritis. Nat. Biotechnol. 31, 142–147 (2013). 23334450 10.1038/nbt.2487 PMC3598632

[b8] Dick K. J. . DNA methylation and body-mass index: a genome-wide analysis. Lancet 383, 1990–1998 (2014). 24630777 10.1016/S0140-6736(13)62674-4

[b9] Maurano M. T. . Systematic localization of common disease-associated variation in regulatory DNA. Science 337, 1190–1195 (2012). 22955828 10.1126/science.1222794 PMC3771521

[b10] Ernst J. . Mapping and analysis of chromatin state dynamics in nine human cell types. Nature 473, 43–49 (2011). 21441907 10.1038/nature09906 PMC3088773

[b11] Ziller M. J. . Charting a dynamic DNA methylation landscape of the human genome. Nature 500, 477–481 (2013). 23925113 10.1038/nature12433 PMC3821869

[b12] Hodges E. . High definition profiling of mammalian DNA methylation by array capture and single molecule bisulfite sequencing. Genome Res. 19, 1593–1605 (2009). 19581485 10.1101/gr.095190.109 PMC2752124

[b13] Li H. & Durbin R. Fast and accurate short read alignment with Burrows–Wheeler transform. Bioinformatics 25, 1754–1760 (2009). 19451168 10.1093/bioinformatics/btp324 PMC2705234

[b14] Marceau P. . Duodenal switch improved standard biliopancreatic diversion: a retrospective study. Surg. Obes. Relat. Dis. 5, 43–47 (2009). 18440876 10.1016/j.soard.2008.03.244

[b15] Expert Panel on Detection, E. Executive summary of the third report of the National Cholesterol Education Program (NCEP) expert panel on Detection, Evaluation, and Treatment of high blood cholesterol in adults (Adult Treatment Panel III). JAMA 285, 2486 (2001). 11368702 10.1001/jama.285.19.2486

[b16] Burger L., Gaidatzis D., Schübeler D. & Stadler M. B. Identification of active regulatory regions from DNA methylation data. Nucleic Acids Res. 41, e155–e155 (2013). 23828043 10.1093/nar/gkt599 PMC3763559

[b17] Fortin J.-P. . Functional normalization of 450k methylation array data improves replication in large cancer studies. bioRxiv 15, 503 (2014). 10.1186/s13059-014-0503-2 PMC4283580 25599564

[b18] Spector T. D. & Williams F. M. The UK adult twin registry (TwinsUK). Twin Res. Hum. Genet. 9, 899–906 (2006). 17254428 10.1375/183242706779462462

[b19] Andrew T. . Are twins and singletons comparable? A study of disease-related and lifestyle characteristics in adult women. Twin Res. 4, 464–477 (2001). 11780939 10.1375/1369052012803

[b20] Buenrostro J. D., Giresi P. G., Zaba L. C., Chang H. Y. & Greenleaf W. J. Transposition of native chromatin for fast and sensitive epigenomic profiling of open chromatin, DNA-binding proteins and nucleosome position. Nat. Methods 10, 1213–1218 (2013). 24097267 10.1038/nmeth.2688 PMC3959825

[b21] Silverstein R. L. & Febbraio M. CD36, a scavenger receptor involved in immunity, metabolism, angiogenesis, and behavior. Sci. Signal. 2, re3 (2009). 19471024 10.1126/scisignal.272re3 PMC2811062

[b22] Love-Gregory L. & Abumrad N. A. CD36 genetics and the metabolic complications of obesity. Curr. Opin. Clin. Nutr. Metab. Care 14, 527 (2011). 21912245 10.1097/MCO.0b013e32834bbac9 PMC3295590

[b23] Rać M. E., Safranow K. & Poncyljusz W. Molecular basis of human CD36 gene mutations. Mol. Med. 13, 288 (2007). 17673938 10.2119/2006-00088.Rac PMC1936231

[b24] Knøsgaard L., Thomsen S., Støckel M., Vestergaard H. & Handberg A. Circulating sCD36 is associated with unhealthy fat distribution and elevated circulating triglycerides in morbidly obese individuals. Nutr. Diabetes 4, e114 (2014). 24710072 10.1038/nutd.2014.11 PMC4007154

[b25] Coram M. A. . Genome-wide characterization of shared and distinct genetic components that influence blood lipid levels in ethnically diverse human populations. Am. J. Hum. Genet. 92, 904–916 (2013). 23726366 10.1016/j.ajhg.2013.04.025 PMC3675231

[b26] Rakyan V. K., Down T. A., Balding D. J. & Beck S. Epigenome-wide association studies for common human diseases. Nat. Rev. Genet. 12, 529–541 (2011). 21747404 10.1038/nrg3000 PMC3508712

[b27] Alkhatatbeh M., Enjeti A., Acharya S., Thorne R. & Lincz L. The origin of circulating CD36 in type 2 diabetes. Nutr. Diabetes 3, e59 (2013). 23381664 10.1038/nutd.2013.1 PMC3584987

[b28] Shen Y. . A map of the cis-regulatory sequences in the mouse genome. Nature 488, 116–120 (2012). 22763441 10.1038/nature11243 PMC4041622

[b29] Johnson M. D., Mueller M., Game L. & Aitman T. J. Single nucleotide analysis of cytosine methylation by whole-genome shotgun bisulfite sequencing. Curr. Protoc. Mol. Biol. 21.23. 21–21.23. 28 (2012). 10.1002/0471142727.mb2123s99 22870857

[b30] Marceau P. . Biliopancreatic diversion with duodenal switch. World. J. Surg. 22, 947–954 (1998). 9717420 10.1007/s002689900498

[b31] Vohl M. C. . A survey of genes differentially expressed in subcutaneous and visceral adipose tissue in men*. Obes. Res. 12, 1217–1222 (2004). 15340102 10.1038/oby.2004.153

[b32] Richterich R. & Dauwalder H. [Determination of plasma glucose by hexokinase-glucose-6-phosphate dehydrogenase method]. Schweiz. Med. Wochenschr. 101, 615–618 (1971). 5576952

[b33] Liu Y., Siegmund K. D., Laird P. W. & Berman B. P. Bis-SNP: combined DNA METHYLATION and SNP calling for bisulfite-seq data. Genome Biol. 13, R61 (2012). 22784381 10.1186/gb-2012-13-7-r61 PMC3491382

[b34] Tchernof A. . Regional differences in adipose tissue metabolism in women minor effect of obesity and body fat distribution. Diabetes 55, 1353–1360 (2006). 16644692 10.2337/db05-1439

[b35] Lohse M. . RobiNA: a user-friendly, integrated software solution for RNA-Seq-based transcriptomics. Nucleic Acids Res. 40, W622–W627 (2012). 22684630 10.1093/nar/gks540 PMC3394330

[b36] Feng J., Liu T., Qin B., Zhang Y. & Liu X. S. Identifying ChIP-seq enrichment using MACS. Nat. Protoc. 7, 1728–1740 (2012). 22936215 10.1038/nprot.2012.101 PMC3868217

[b37] Guenard F. . Association of LIPA gene polymorphisms with obesity-related metabolic complications among severely obese patients. Obesity 20, 2075–2082 (2012). 22395809 10.1038/oby.2012.52

[b38] Turcot V. . LINE-1 methylation in visceral adipose tissue of severely obese individuals is associated with metabolic syndrome status and related phenotypes. Clin. Epigenet. 4, 10 (2012). 10.1186/1868-7083-4-10 PMC3464682 22748066

[b39] Trapnell C., Pachter L. & Salzberg S. L. TopHat: discovering splice junctions with RNA-Seq. Bioinformatics 25, 1105–1111 (2009). 19289445 10.1093/bioinformatics/btp120 PMC2672628

[b40] Langmead B., Trapnell C., Pop M. & Salzberg S. L. Ultrafast and memory-efficient alignment of short DNA sequences to the human genome. Genome Biol. 10, R25 (2009). 19261174 10.1186/gb-2009-10-3-r25 PMC2690996

[b41] Anders S. & Huber W. Differential expression analysis for sequence count data. Genome Biol. 11, R106 (2010). 20979621 10.1186/gb-2010-11-10-r106 PMC3218662

