# Supplementary material for: Characterization of functional methylomes by next-generation capture sequencing identifies novel disease-associated variants
Source: Nat Commun. 2015 May 29;6:7211. doi: 10.1038/ncomms8211 (PMC4544751; doi:10.1038/ncomms8211)
Supplement: Supplementary Information — Supplementary Figures 1-11 and Supplementary Tables 1-7 [file ncomms8211-s1.pdf]

Supplementary Figures

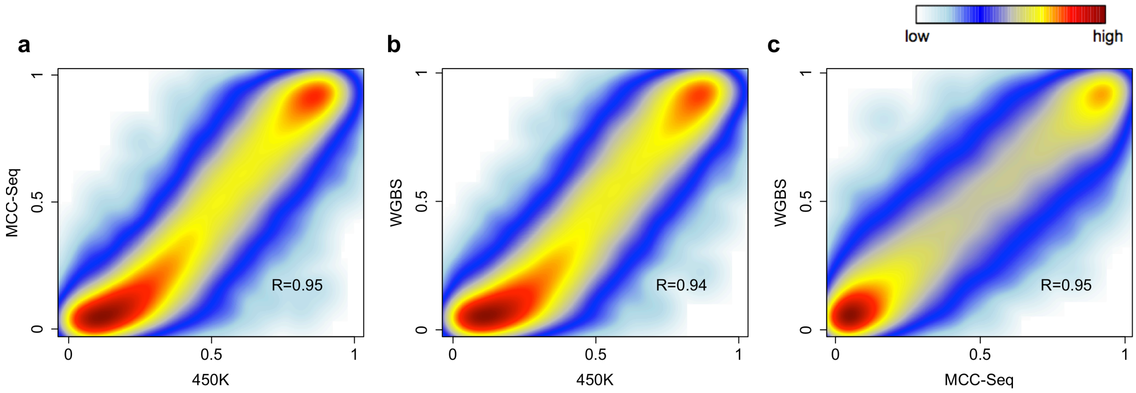

Supplementary Figure 1. Extended comparison of MCC-Seq methylation calls with WGBS and the Illumina 450K array excluding completely hypo and hypermethylated CpGs (**a**) Correlation between MCC-Seq$_{4\text{-plex}}$ and Illumina 450K array methylation calls for the same VAT DNA sample (R=0.95), (**b**) comparison between WGBS and Illumina 450K array results (R=0.94) and (**c**) comparison between WGBS and MCC-Seq$_{4\text{-plex}}$ results (R=0.95). Only CpGs with data available from all three techniques were included as well as excluding completely hypo and hypermethylated CpGs (N=150,898 CpGs); we required sequence coverage ≥5X for MCC-Seq and WGBS; R is the Pearson correlation coefficient.

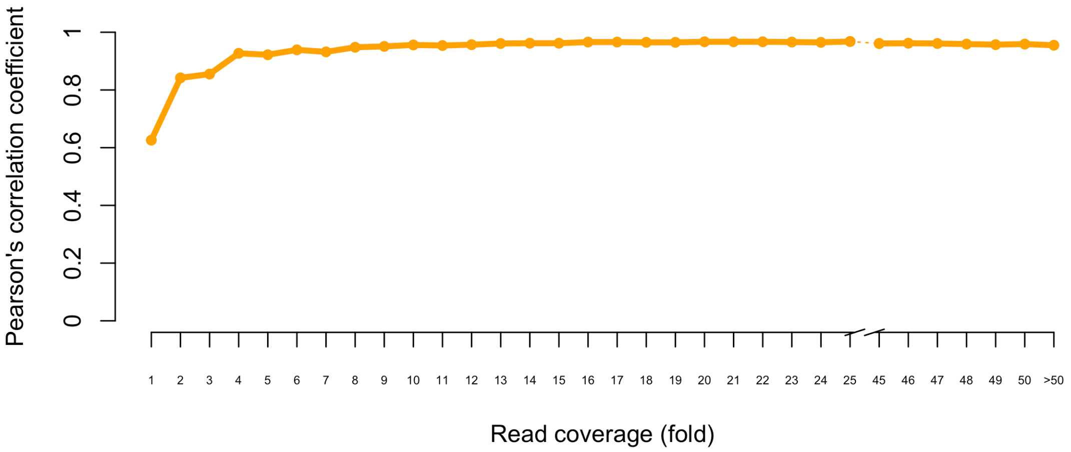

Supplementary Figure 2. Correlation between Illumina 450K array and MCC-Seq methylation calls at different read coverage Pearson correlation between Illumina 450K array and MCC-Seq methylation calls for the same VAT DNA sample is shown at different read depth (fold, x-axis).

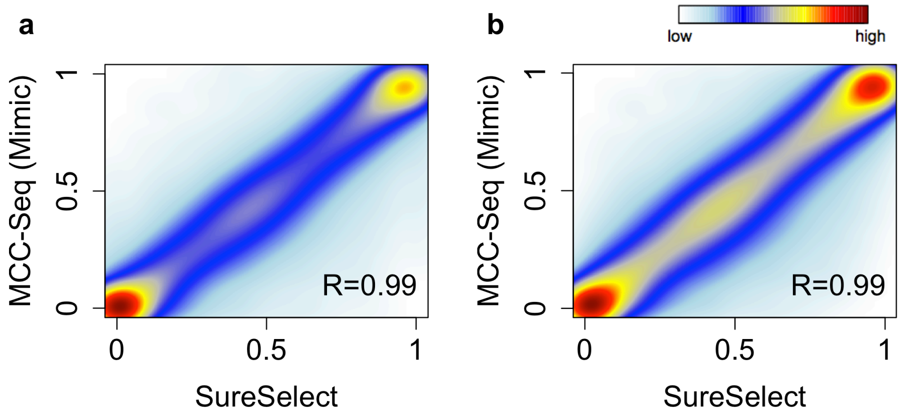

Supplementary Figure 3. Comparison of MCC-Seq methylation calls with Agilent SureSelect Correlations between MCC-Seq$_{Mimic}$ and Agilent SureSelect methylation calls for the LCL GM12878 cell line (**a**) using all overlapping CpGs (N=2,551,186; R=0.99) and (**b**) excluding hypo (0%) and hypermethylated CpGs (N=1,734,371; R=0.99). Only CpGs with data available from both techniques were included; we required sequence coverage ≥5X for MCC-Seq and WGBS accordingly; R is the Pearson correlation coefficient; density scales are independent in the two panels.

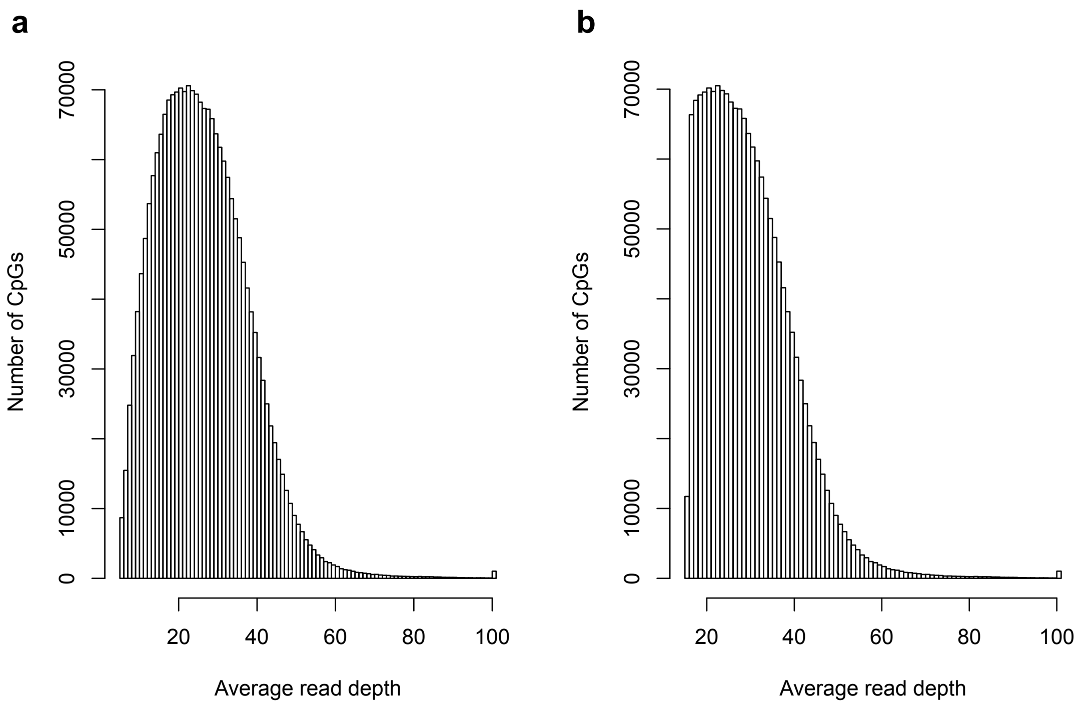

Supplementary Figure 4. Distributions of sequence coverage at included CpG sites Distributions of the average read depth across the 72 individuals of included CpG sites when considering (**a**) all CpG sites with ≥5X and ≤ 100X sequence depth and (**b**) sites meeting these criteria and with average sequence depth above the 20th percentile.

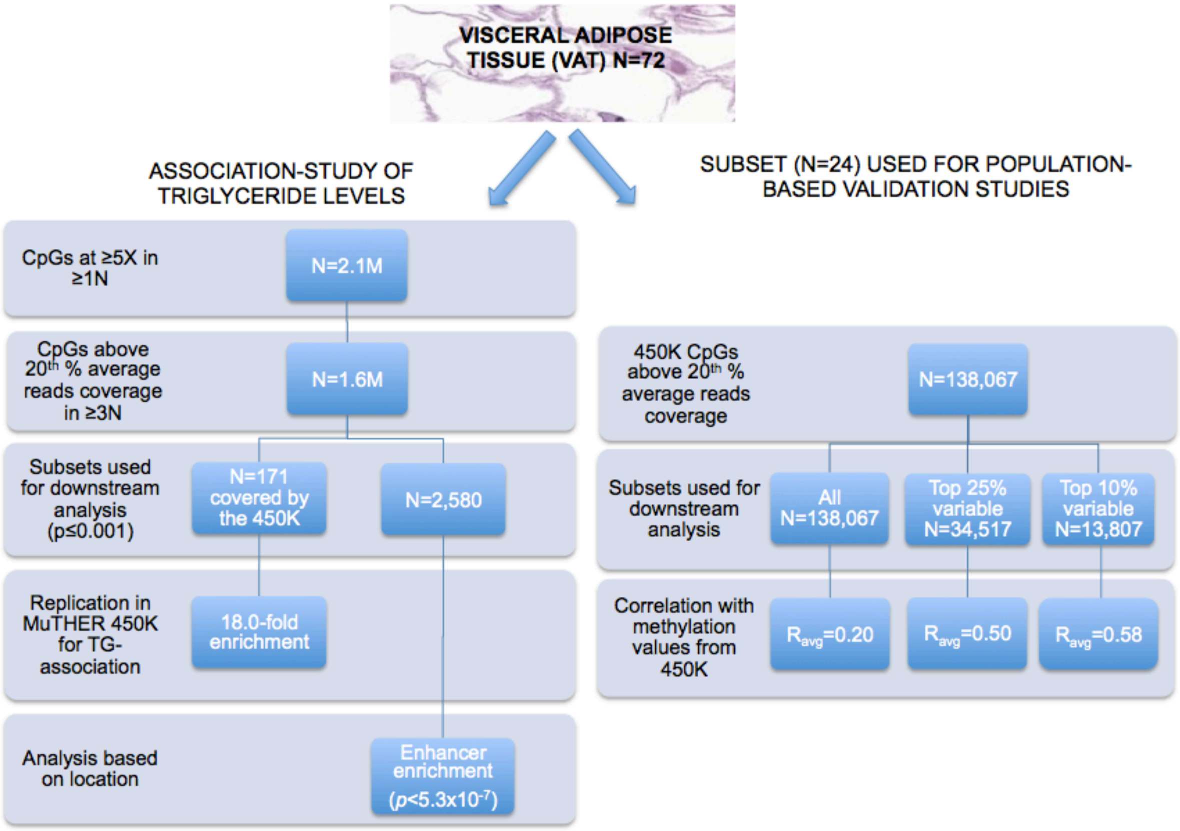

Supplementary Figure 5. Outline of the trait association and population-based validation studies In the left panel, an outline of the subsets used for different analysis in the association-study of methylation to triglyceride levels as well as main results are presented. In the right panel, the outline of the population-based validation studies comparing methylation values obtained with the 450K array and MCC-Seq in 24 individuals is shown.

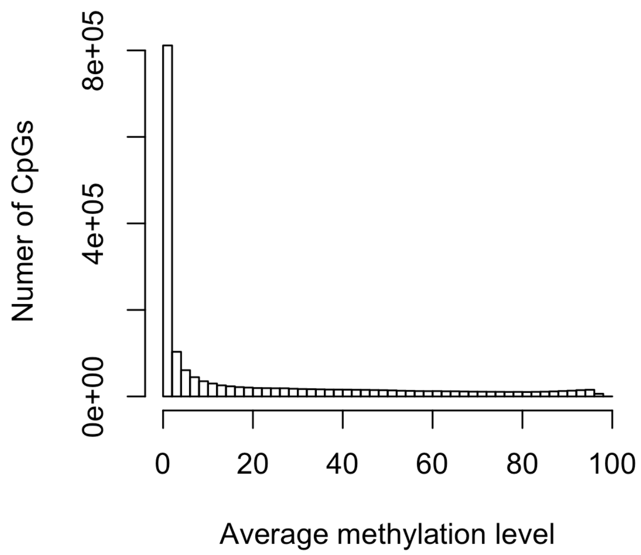

Supplementary Figure 6. Average methylation pattern of CpGs captured with MCC-Seq Met V1 design The figure shows the average methylation values (%, x-axis) for 72 visceral adipose tissue samples at on-target CpG sites above the 20th percentile average reads coverage (N=1,710,209) from Met V1 capture experiments.

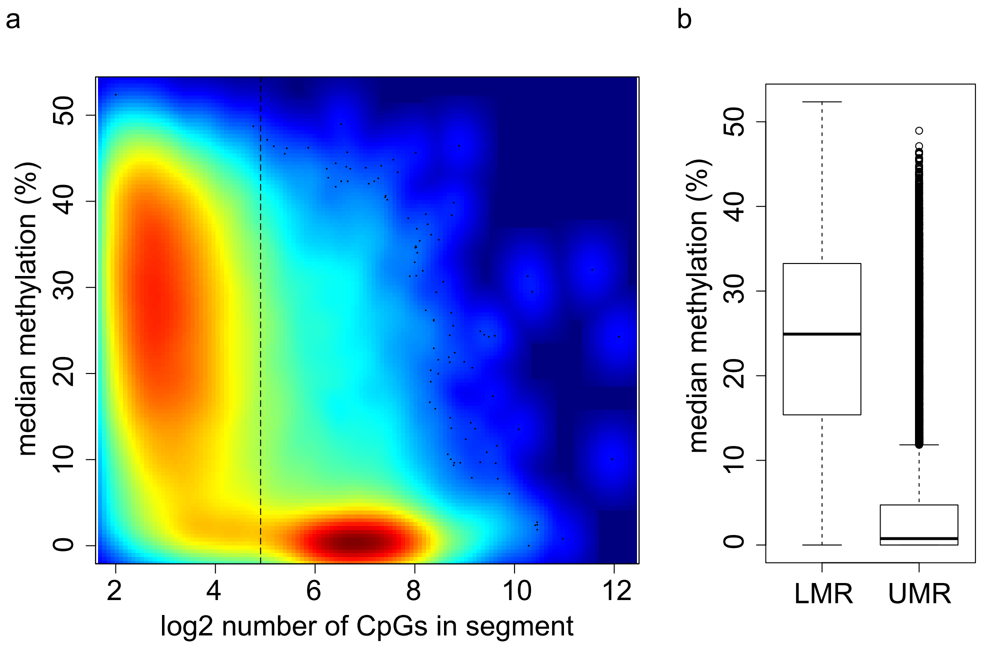

Supplementary Figure 7. Characterization of adipose hypomethylated footprints Hypomethylated footprints were generated from WGBS on AT using the R/Bioconductor package MethylSeekR. (**a**) Unmethylated regions (UMRs, right of dotted line) and low-methylated regions (LMRs, left of dotted line) were differentiated by a 30 CpG content threshold. (**b**) LMRs (N=45,065) and UMRs (N=20,195) show different median methylation patterns with LMRs having a broader range of methylation and UMRs being less variable in their methylation status and associated to low-methylated promoter regions. Boxplot whiskers represent 1.5*IQR (inter quartile range).

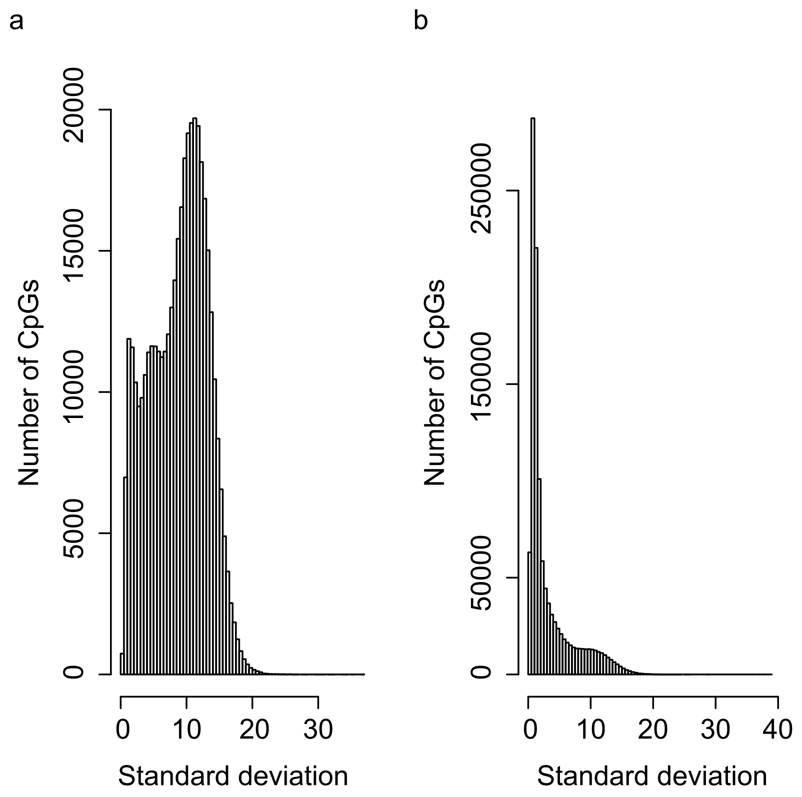

Supplementary Figure 8. Variability of enhancer and promoter associated CpG sites Interrogated CpG sites were mapped to putative enhancers (H3K4me1 or LMR) or promoters (H3K4me3 and UMR). Assessing the standard deviation of the methylation status across individuals (**a**) CpGs mapping to putative enhancers were found to be more variable (median SD=9.4) than (**b**) those mapping to putative promoters (median SD=1.5).

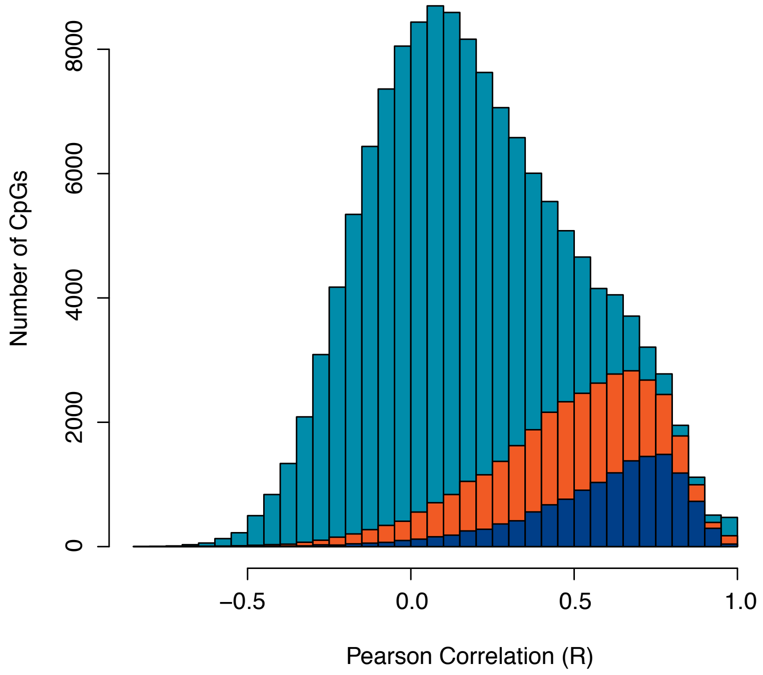

Supplementary Figure 9. CpG-by-CpG correlation between Illumina 450K array and MCC-Seq methylation calls in 24 samples The figure shows the distribution of spearman rank correlations for CpGs from the Illumina 450K array that had average reads coverage above the 20th percentile in the MCC-Seq method (teal; N=138,067 CpGs; mean R =0.20). The correlations were generally greater when we included only the top 25% variable CpGs (orange; N=34,517 CpGs; mean R=0.50) or the top 10% variable CpGs (blue; N=13,807 CpGs; mean R=0.58) from the MCC-Seq methylation calls.

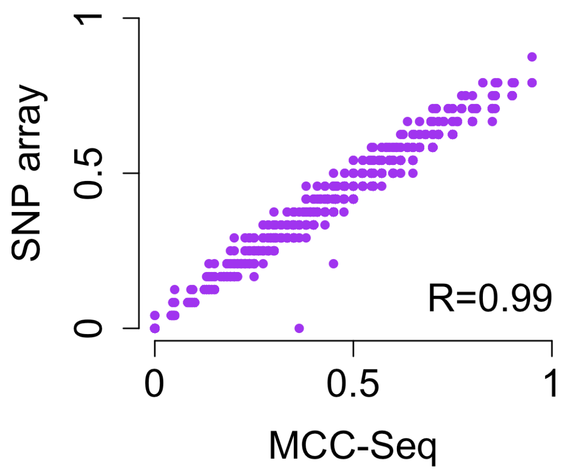

Supplementary Figure 10. Comparison of the observed heterozygosity from MCC-Seq and HumanOmni BeadChip array genotyping calls Comparison between the observed heterozygosity based on the HumanOmni BeadChip array (SNP ratio, y-axis) and MCC-Seq$_{4\text{-plex}}$ (SNP ratio, x-axis) genotyping calls across ≥20 individuals (N=3,093 SNPs; R=0.99); we required sequence coverage ≥5X for MCC-Seq; R is the Pearson correlation coefficient.

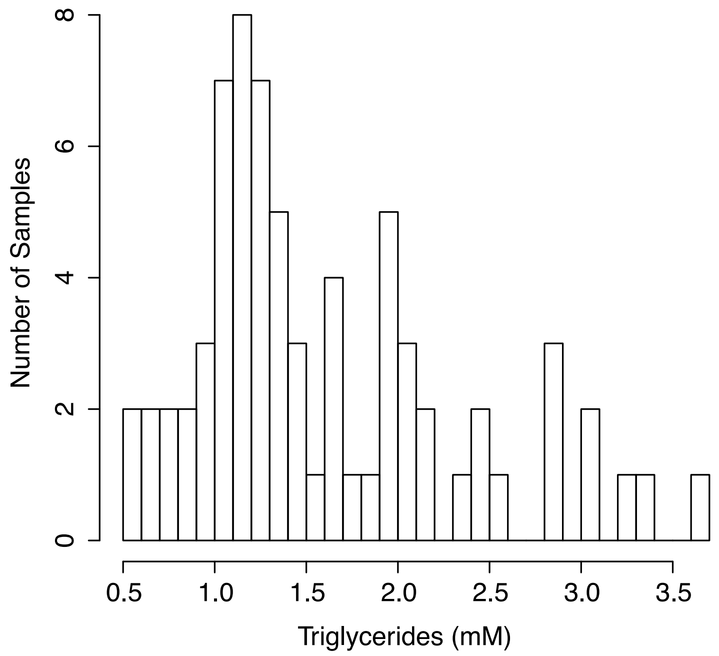

Supplementary Figure 11. Distribution of triglycerides levels in the discovery cohort Distributions of the triglyceride levels (mM, x-axis) across the discovery cohort (N=70, two outliers excluded).

Supplementary Table 1. Sequence statistics of the Met V1 pooled samples

	Plexing level	Sample ID	raw reads	Total aligned reads (%)	Total on-target reads (%)	Total on-target aligned reads (%)	On-target aligned reads	Average coverage on-target CpG sites	Total captured on-target CpG sites (%)	Total CpG sites with >=5 reads (%)	Total CpG sites with >=10 reads (%)
Individual	1	1	257,885,682	60	67	40	95,832,520	82	99	94	94
	2	1	151,069,388	60	71	43	58,871,218	53	99	91	90
	2	2	144,059,126	62	69	43	57,386,604	50	99	90	89
	4	3	65,799,220	70	79	55	35,355,720	33	99	82	80
	4	4	62,929,502	72	80	57	34,630,762	33	99	82	79
	4	5	57,375,348	72	79	57	31,638,544	30	99	80	77
	4	6	51,172,122	73	78	57	28,258,060	27	99	80	76
	6	1	65,153,260	74	55	41	26,090,152	24	99	80	76
	6	2	51,084,062	76	56	43	21,392,302	21	99	77	71
	6	7	59,331,638	73	55	40	23,132,120	21	99	77	71
	6	8	72,222,874	74	55	41	29,161,380	27	99	82	78
	6	9	65,481,242	73	54	39	25,251,428	24	99	80	75
	6	10	71,864,932	72	54	39	27,650,522	25	99	80	76
	10	11	36,474,056	75	59	44	15,739,422	14	98	65	54
	10	12	32,548,084	76	59	45	14,374,870	13	98	63	51
	10	13	32,058,534	76	59	45	14,139,984	13	98	62	49
	10	14	29,780,130	75	60	45	13,047,378	12	98	60	46
	10	15	38,922,258	74	58	43	16,445,576	15	98	67	56
	10	16	34,623,254	74	57	42	14,442,798	13	98	62	50
	10	17	40,850,638	75	51	38	15,457,642	14	98	66	55
	10	18	28,196,414	74	58	43	11,877,752	10	98	55	38
	10	19	31,954,644	76	60	46	14,209,644	13	98	63	50
	10	20	32,427,388	75	59	44	14,171,532	13	98	62	49
Average	1	NA	257,885,682	60	67	40	95,832,520	82	99	94	94
	2	NA	147,564,257	61	70	43	58,128,911	51	99	90	90
	4	NA	59,319,048	72	79	57	31,194,648	31	99	81	78
	6	NA	64,189,668	74	55	41	25,446,317	24	99	79	75
	10	NA	33,783,540	75	58	44	14,390,660	13	98	63	50

Supplementary Table 2. Sequence statistics of the Met V2 pooled samples

	Plexing level	Sample ID	raw reads	Total aligned reads (%)	Total on-target reads (%)	Total on-target aligned reads (%)	On-target aligned reads	Average coverage on-target CpG sites	Total captured on-target CpG sites (%)	Total CpG sites with >=5 reads (%)	Total CpG sites with >=10 reads (%)
Individual	6	21	52,965,668	71	62	44	23,013,966	13	98	62	50
	6	22	52,005,852	72	62	44	22,681,612	13	98	62	50
	6	23	76,717,544	73	62	45	34,152,304	20	98	73	67
	6	24	59,006,738	73	61	45	26,096,106	15	98	65	54
	6	25	53,915,196	71	62	44	23,447,444	14	98	63	51
	6	26	60,990,908	72	63	45	27,036,892	16	98	67	58
**Average**	6	NA	59,266,984	72	62	45	26,071,387	15	98	65	55

Supplementary Table 3. Comparison of MCC-Seq methylation calls with Illumina 450K array and WGBS data at various read depths

Technique comparisons	Pearson Correlation			
	>=5X (N=150,898 CpGs)	>=10X (N=144,868 CpGs)	>=20X (N=90,547 CpGs)	>=30X (N=17,852 CpGs)
**450K~MCC-Seq**	0.964	0.964	0.965	0.962
**450K~WGBS**	0.962	0.962	0.961	0.959
**MCC-Seq~WGBS**	0.974	0.974	0.974	0.972

Supplementary Table 4. Comparison of MCC-Seq methylation calls with Illumina 450K array and WGBS data at various read depths excluding completely hypo and hypermethylated CpGs

Technique comparisons	Pearson Correlation			
	>=5X (N=45,097 CpGs)	>=10X (N=44,414 CpGs)	>=20X (N=30,934 CpGs)	>=30X (N=7,182 CpGs)
**450K~MCC-Seq**	0.946	0.947	0.952	0.953
**450K~WGBS**	0.942	0.943	0.946	0.947
**MCC-Seq~WGBS**	0.949	0.950	0.955	0.958

Supplementary Table 5. MuTHER replication and cis-mQTL regulation of top TG-associated CpGs

CpG rank	CpG chr	CpG start	CpG end	450K ProbeID	450K probe start position	450K probe end postion	MuTHER Anova association p-value	MuTHER beta value	MuTHER replication status	SNP	SNP position	MAF	cis-mQTL p-value	cis-mQTL beta value	Gene	cis-mQTL status at FDR 1%
1	chr7	80276086	80276087	cg05917188	80275870	80275919	3.22E-07	0.02160	REPLICATED	rs7755	80306271	0.444	1.52E-01	-0.00522	CD36	.
2	chr18	46459491	46459492	NA	NA	NA	NA	NA	NA	NA	NA	NA	NA	NA	NA	NA
3	chr14	82002624	82002625	cg25066972	82002568	82002617	1.47E-08	0.02701	REPLICATED	rs7149751	82042759	0.182	7.40E-04	0.01867	NA	SIGNIFICANT
4	chr15	40374156	40374157	cg11448015	40374034	40374083	1.16E-01	-0.00098	.	rs998713	40378467	0.401	4.13E-02	0.00143	NA	.
5	chr12	68352059	68352060	NA	NA	NA	NA	NA	NA	NA	NA	NA	NA	NA	NA	NA
6	chr10	14791641	14791642	NA	NA	NA	NA	NA	NA	NA	NA	NA	NA	NA	NA	NA
7	chr17	78747573	78747574	NA	NA	NA	NA	NA	NA	NA	NA	NA	NA	NA	NA	NA
8	chr17	70419164	70419165	NA	NA	NA	NA	NA	NA	NA	NA	NA	NA	NA	NA	NA
9	chr7	2527288	2527289	cg07446753	2527391	2527440	1.24E-06	0.01593	REPLICATED	rs10488610	2606934	0.074	3.20E-04	-0.01873	NA	SIGNIFICANT
10	chr7	105494830	105494831	NA	NA	NA	NA	NA	NA	NA	NA	NA	NA	NA	NA	NA
11	chr14	93499058	93499059	NA	NA	NA	NA	NA	NA	NA	NA	NA	NA	NA	NA	NA
12	chr11	75786020	75786021	NA	NA	NA	NA	NA	NA	NA	NA	NA	NA	NA	NA	NA
13	chr17	78747529	78747530	NA	NA	NA	NA	NA	NA	NA	NA	NA	NA	NA	NA	NA
14	chr4	127837946	127837947	NA	NA	NA	NA	NA	NA	NA	NA	NA	NA	NA	NA	NA
15	chr15	91006854	91006855	NA	NA	NA	NA	NA	NA	NA	NA	NA	NA	NA	NA	NA
16	chr1	1174212	1174213	cg00657094	1174190	1174239	1.37E-01	0.00441	.	rs3813199	1158277	0.102	1.10E-34	0.05102	NA	SIGNIFICANT
17	chr6	74276861	74276862	cg04958236	74276973	74277022	2.28E-01	-0.00369	.	rs4708064	74275405	0.338	3.56E-32	0.03455	NA	SIGNIFICANT
18	chr9	34892821	34892822	NA	NA	NA	NA	NA	NA	NA	NA	NA	NA	NA	NA	NA
19	chr9	80055277	80055278	NA	NA	NA	NA	NA	NA	NA	NA	NA	NA	NA	NA	NA
20	chr1	10895564	10895565	cg17480077	10895517	10895566	8.17E-01	-0.00058	.	rs1292657	10893081	0.233	1.04E-06	0.01362	NA	SIGNIFICANT
21	chr3	193988521	193988522	cg16932827	193988639	193988688	5.02E-08	0.01964	REPLICATED	rs7651474	193943677	0.389	1.95E-02	-0.00765	NA	.
22	chr21	44739256	44739257	NA	NA	NA	NA	NA	NA	NA	NA	NA	NA	NA	NA	NA
23	chr2	65576937	65576938	NA	NA	NA	NA	NA	NA	NA	NA	NA	NA	NA	NA	NA
24	chr17	41003976	41003977	NA	NA	NA	NA	NA	NA	NA	NA	NA	NA	NA	NA	NA
25	chr1	224921325	224921326	NA	NA	NA	NA	NA	NA	NA	NA	NA	NA	NA	NA	NA
26	chr14	101985031	101985032	NA	NA	NA	NA	NA	NA	NA	NA	NA	NA	NA	NA	NA
27	chr17	27439437	27439438	NA	NA	NA	NA	NA	NA	NA	NA	NA	NA	NA	NA	NA
28	chr18	77246010	77246011	cg18935516	77245874	77245923	2.08E-01	-0.00324	.	rs1562724	77169571	0.112	4.62E-03	0.01148	NFATC1	.
29	chr9	33140269	33140270	cg13858803	33140269	33140318	4.87E-05	0.01299	SUGGESTED	rs1013217	33104540	0.259	3.49E-03	-0.00908	B4GALT1	.
30	chr10	88013023	88013024	cg24351003	88013210	88013259	8.09E-01	-0.00050	.	rs2814352	88014349	0.166	4.92E-05	0.01005	GRID1	SIGNIFICANT
31	chr4	4398066	4398067	cg06860998	4398094	4398143	8.48E-02	0.00479	.	rs4555567	4397537	0.265	3.20E-30	0.03152	NSG1	SIGNIFICANT
32	chr6	55533610	55533611	NA	NA	NA	NA	NA	NA	NA	NA	NA	NA	NA	NA	NA
33	chr16	85158006	85158007	cg11201751	85158047	85158096	3.99E-08	0.01938	REPLICATED	rs2062550	85164601	0.452	2.38E-06	-0.01478	NA	SIGNIFICANT
34	chr7	37252350	37252351	NA	NA	NA	NA	NA	NA	NA	NA	NA	NA	NA	NA	NA
35	chr20	60932998	60932999	NA	NA	NA	NA	NA	NA	NA	NA	NA	NA	NA	NA	NA
36	chr1	205228721	205228722	NA	NA	NA	NA	NA	NA	NA	NA	NA	NA	NA	NA	NA
37	chr16	2089530	2089531	NA	NA	NA	NA	NA	NA	NA	NA	NA	NA	NA	NA	NA
38	chr19	47129789	47129790	cg04706667	47129637	47129686	6.95E-05	0.00737	SUGGESTED	rs11083841	47120033	0.153	6.91E-04	0.00780	PTGIR	SIGNIFICANT
39	chr13	59931088	59931089	NA	NA	NA	NA	NA	NA	NA	NA	NA	NA	NA	NA	NA
40	chr5	34496834	34496835	NA	NA	NA	NA	NA	NA	NA	NA	NA	NA	NA	NA	NA
41	chr16	57825356	57825357	NA	NA	NA	NA	NA	NA	NA	NA	NA	NA	NA	NA	NA
42	chr6	6656625	6656626	NA	NA	NA	NA	NA	NA	NA	NA	NA	NA	NA	NA	NA
43	chr13	40957432	40957433	NA	NA	NA	NA	NA	NA	NA	NA	NA	NA	NA	NA	NA
44	chr4	7825455	7825456	NA	NA	NA	NA	NA	NA	NA	NA	NA	NA	NA	NA	NA
45	chr15	31695119	31695120	NA	NA	NA	NA	NA	NA	NA	NA	NA	NA	NA	NA	NA
46	chr4	2808262	2808263	cg10869531	2808489	2808538	1.55E-02	0.00591	.	rs1269392	2783440	0.162	2.23E-05	-0.01478	SH3BP2	SIGNIFICANT
47	chr22	18042778	18042779	cg04460557	18042646	18042695	1.36E-01	0.00325	.	rs174351	18038786	0.323	1.42E-05	-0.00909	SLC25A18	SIGNIFICANT
48	chr12	118227823	118227824	NA	NA	NA	NA	NA	NA	NA	NA	NA	NA	NA	NA	NA
49	chr16	88351337	88351338	NA	NA	NA	NA	NA	NA	NA	NA	NA	NA	NA	NA	NA
50	chr19	584401	584402	cg26956799	584425	584474	3.97E-01	0.00171	.	rs3814895	588860	0.262	2.01E-03	-0.00623	NA	.
51	chr9	137258822	137258823	cg13786567	137258872	137258921	3.93E-01	-0.00149	.	rs6537944	137312828	0.078	2.35E-02	0.00717	RXRA	.
52	chr1	235054358	235054359	NA	NA	NA	NA	NA	NA	NA	NA	NA	NA	NA	NA	NA
53	chr15	23892836	23892837	cg04142555	23892608	23892657	9.67E-02	0.00317	.	rs7495382	23824746	0.222	6.04E-02	0.00412	MAGEL2	.
54	chr3	61720610	61720611	NA	NA	NA	NA	NA	NA	NA	NA	NA	NA	NA	NA	NA
55	chr7	148661205	148661206	NA	NA	NA	NA	NA	NA	NA	NA	NA	NA	NA	NA	NA
56	chr5	54818087	54818088	NA	NA	NA	NA	NA	NA	NA	NA	NA	NA	NA	NA	NA
57	chr22	37678241	37678242	cg19751930	37678222	37678271	3.09E-02	-0.00411	.	rs6000649	37677490	0.45	9.63E-20	0.01607	CYTH4	SIGNIFICANT
58	chr3	124555885	124555886	NA	NA	NA	NA	NA	NA	NA	NA	NA	NA	NA	NA	NA
59	chr7	27179312	27179313	cg17775899	27179378	27179427	4.18E-02	0.00449	SUGGESTED	rs3757640	27182245	0.255	7.36E-03	0.00660	NA	.
60	chr17	77404010	77404011	NA	NA	NA	NA	NA	NA	NA	NA	NA	NA	NA	NA	NA
61	chr2	38387408	38387409	cg10481134	38387395	38387444	7.48E-05	0.01214	SUGGESTED	rs4670835	38469162	0.156	6.05E-04	0.01211	CYP1B1-AS	SIGNIFICANT
62	chr5	141564812	141564813	NA	NA	NA	NA	NA	NA	NA	NA	NA	NA	NA	NA	NA
63	chr5	31213788	31213789	NA	NA	NA	NA	NA	NA	NA	NA	NA	NA	NA	NA	NA
64	chr3	38765465	38765466	NA	NA	NA	NA	NA	NA	NA	NA	NA	NA	NA	NA	NA
65	chr7	30954512	30954513	cg03310518	30954474	30954523	1.87E-02	-0.00668	.	rs10225302	31001075	0.348	7.73E-02	-0.00558	AQP1	.
66	chr22	33105136	33105137	NA	NA	NA	NA	NA	NA	NA	NA	NA	NA	NA	NA	NA
67	chr2	233764015	233764016	NA	NA	NA	NA	NA	NA	NA	NA	NA	NA	NA	NA	NA
68	chr2	76895256	76895257	NA	NA	NA	NA	NA	NA	NA	NA	NA	NA	NA	NA	NA
69	chr22	45947785	45947786	NA	NA	NA	NA	NA	NA	NA	NA	NA	NA	NA	NA	NA
70	chr13	40957443	40957444	NA	NA	NA	NA	NA	NA	NA	NA	NA	NA	NA	NA	NA
71	chr6	166263737	166263738	cg06738040	166263737	166263786	7.83E-05	-0.01385	SUGGESTED	rs9348049	166263379	0.426	2.58E-04	-0.01104	NA	SIGNIFICANT
72	chr15	31355481	31355482	cg20640374	31355304	31355353	4.20E-07	0.01879	REPLICATED	rs6493454	31393945	0.423	1.62E-02	-0.00811	TRPM1	.
73	chr10	131748428	131748429	cg02449166	131748481	131748530	7.12E-03	0.00277	SUGGESTED	rs4751144	131702075	0.286	1.32E-02	0.00253	EBF3	.
74	chr1	21819101	21819102	cg13170600	21818815	21818864	4.03E-03	0.00580	SUGGESTED	rs12082914	21819738	0.222	8.96E-06	-0.00990	NA	SIGNIFICANT
75	chr8	142309659	142309660	cg10090414	142309604	142309653	2.03E-06	0.01473	REPLICATED	rs11781174	142297744	0.337	2.51E-02	0.00688	NA	.
76	chr9	34559411	34559412	NA	NA	NA	NA	NA	NA	NA	NA	NA	NA	NA	NA	NA
77	chr5	176091609	176091610	cg25386845	176091428	176091477	6.12E-01	0.00089	.	rs4868684	176091730	0.35	3.34E-16	-0.01283	NA	SIGNIFICANT
78	chr2	44046117	44046118	NA	NA	NA	NA	NA	NA	NA	NA	NA	NA	NA	NA	NA
79	chrX	45557089	45557090	NA	NA	NA	NA	NA	NA	NA	NA	NA	NA	NA	NA	NA
80	chr22	36721648	36721649	cg09051119	36721648	36721697	1.11E-01	0.00282	.	rs11704382	36728152	0.06	1.51E-02	0.00842	MYH9	.
81	chr14	105670861	105670862	cg23568737	105670754	105670803	1.91E-01	0.00209	.	rs8020454	105685865	0.293	1.27E-11	0.00999	NA	SIGNIFICANT
82	chr3	185701636	185701637	NA	NA	NA	NA	NA	NA	NA	NA	NA	NA	NA	NA	NA
83	chr10	102567936	102567937	NA	NA	NA	NA	NA	NA	NA	NA	NA	NA	NA	NA	NA
84	chr11	125386885	125386886	cg18493761	125386837	125386886	2.76E-02	0.00545	SUGGESTED	rs665390	125347506	0.224	1.42E-08	0.01471	NA	SIGNIFICANT
85	chr12	123402648	123402649	NA	NA	NA	NA	NA	NA	NA	NA	NA	NA	NA	NA	NA
86	chr8	22674602	22674603	NA	NA	NA	NA	NA	NA	NA	NA	NA	NA	NA	NA	NA
87	chr22	49701234	49701235	cg25569840	49701234	49701283	4.92E-03	0.00338	SUGGESTED	rs13053943	49622092	0.062	1.71E-02	-0.00664	NA	.
88	chr6	75876977	75876978	NA	NA	NA	NA	NA	NA	NA	NA	NA	NA	NA	NA	NA
89	chr18	76481555	76481556	NA	NA	NA	NA	NA	NA	NA	NA	NA	NA	NA	NA	NA

Supplementary Table 6. ATAC-Seq PCR amplification primers

ID	Adaptor Sequence	Primer Sequence
Ad1	n/a	AATGATACGGCGACCACCGAGATCTACACTCGTCGGCAGCGTCAGATGTG
Ad2.1	TAAGGCGA	CAAGCAGAAGACGGCATACGAGATTCGCCTTAGTCTCGTGGGCTCGGAGATGT
Ad2.2	CGTACTAG	CAAGCAGAAGACGGCATACGAGATCTAGTACGGTCTCGTGGGCTCGGAGATGT
Ad2.3	AGGCAGAA	CAAGCAGAAGACGGCATACGAGATTTCTGCCTGTCTCGTGGGCTCGGAGATGT
Ad2.4	TCCTGAGC	CAAGCAGAAGACGGCATACGAGATGCTCAGGAGTCTCGTGGGCTCGGAGATGT
Ad2.5	GGACTCCT	CAAGCAGAAGACGGCATACGAGATAGGAGTCCGTCTCGTGGGCTCGGAGATGT
Ad2.6	TAGGCATG	CAAGCAGAAGACGGCATACGAGATCATGCCTAGTCTCGTGGGCTCGGAGATGT
Ad2.7	CTCTCTAC	CAAGCAGAAGACGGCATACGAGATGTAGAGAGGTCTCGTGGGCTCGGAGATGT
Ad2.8	CAGAGAGG	CAAGCAGAAGACGGCATACGAGATCCTCTCTGGTCTCGTGGGCTCGGAGATGT
Ad2.9	GCTACGCT	CAAGCAGAAGACGGCATACGAGATAGCGTAGCGTCTCGTGGGCTCGGAGATGT
Ad2.10	CGAGGCTG	CAAGCAGAAGACGGCATACGAGATCAGCCTCGGTCTCGTGGGCTCGGAGATGT
Ad2.11	AAGAGGCA	CAAGCAGAAGACGGCATACGAGATTGCCTCTTGTCTCGTGGGCTCGGAGATGT
Ad2.12	GTAGAGGA	CAAGCAGAAGACGGCATACGAGATTCCTCTACGTCTCGTGGGCTCGGAGATGT
Ad2.13	GTCGTGAT	CAAGCAGAAGACGGCATACGAGATATCACGACGTCTCGTGGGCTCGGAGATGT
Ad2.14	ACCACTGT	CAAGCAGAAGACGGCATACGAGATACAGTGGTGTCTCGTGGGCTCGGAGATGT
Ad2.15	TGGATCTG	CAAGCAGAAGACGGCATACGAGATCAGATCCAGTCTCGTGGGCTCGGAGATGT
Ad2.16	CCGTTTGT	CAAGCAGAAGACGGCATACGAGATACAAACGGGTCTCGTGGGCTCGGAGATGT
Ad2.17	TGCTGGGT	CAAGCAGAAGACGGCATACGAGATACCCAGCAGTCTCGTGGGCTCGGAGATGT
Ad2.18	GAGGGGTT	CAAGCAGAAGACGGCATACGAGATAACCCCTCGTCTCGTGGGCTCGGAGATGT
Ad2.19	AGGTTGGG	CAAGCAGAAGACGGCATACGAGATCCCAACCTGTCTCGTGGGCTCGGAGATGT
Ad2.20	GTGTGGTG	CAAGCAGAAGACGGCATACGAGATCACCACACGTCTCGTGGGCTCGGAGATGT
Ad2.21	TGGGTTTC	CAAGCAGAAGACGGCATACGAGATGAAACCCAGTCTCGTGGGCTCGGAGATGT
Ad2.22	TGGTCACA	CAAGCAGAAGACGGCATACGAGATTGTGACCAGTCTCGTGGGCTCGGAGATGT
Ad2.23	TTGACCCT	CAAGCAGAAGACGGCATACGAGATAGGGTCAAGTCTCGTGGGCTCGGAGATGT
Ad2.24	CCACTCCT	CAAGCAGAAGACGGCATACGAGATAGGAGTGGGTCTCGTGGGCTCGGAGATGT

Supplementary Table 7. ATAC-Seq Q-PCR primers

ID	Sequence
Mit_+ve_F	CTAAATAGCCCACACGTTCCC
Mit_+ve_R	AGAGCTCCCGTGAGTGGTTA
GAPDH_+ve_F	CTGTCCCTTCAGTAGCTGCC
GAPDH_+ve_R	GAAGAGAGTGGGTTGGTGGG
GAPDH_-ve_F	TCTGGATGGCCTGAAGGAGA
GAPDH_-ve_R	GCCAGCAGCACTCATGTTTC
ACTB_+ve_F	GAGTCCTTAGGCCGCCAG
ACTB_+ve_R	TCCGACCAGTGTTTGCCTTT
ACTB_-ve_F	CATCTCGTGTCCAGTGCAGA
ACTB_-ve_R	CCATGCAATGTGGGAGTCCT
